# Ontology-Based Combinatorial Comparative Analysis of Adverse Events Associated with Killed and Live Influenza Vaccines

**DOI:** 10.1371/journal.pone.0049941

**Published:** 2012-11-28

**Authors:** Sirarat Sarntivijai, Zuoshuang Xiang, Kerby A. Shedden, Howard Markel, Gilbert S. Omenn, Brian D. Athey, Yongqun He

**Affiliations:** 1 National Center for Integrative Biomedical Informatics, University of Michigan, Ann Arbor, Michigan, United States of America; 2 Center for Computational Medicine and Bioinformatics, University of Michigan, Ann Arbor, Michigan, United States of America; 3 Unit of Laboratory Animal Medicine, University of Michigan Medical School, Ann Arbor, Michigan, United States of America; 4 Department of Microbiology and Immunology, University of Michigan Medical School, Ann Arbor, Michigan, United States of America; 5 Biostatistics Department, School of Public Health, University of Michigan, Ann Arbor, Michigan, United States of America; 6 Center for the History of Medicine and Department of Pediatrics, University of Michigan, Ann Arbor, Michigan, United States of America; 7 Department of Psychiatry, University of Michigan, Ann Arbor, Michigan, United States of America; 8 Departments of Internal Medicine and Human Genetics, and School of Public Health, University of Michigan, Ann Arbor, Michigan, United States of America; Public Health Agency of Canada, Canada

## Abstract

Vaccine adverse events (VAEs) are adverse bodily changes occurring after vaccination. Understanding the adverse event (AE) profiles is a crucial step to identify serious AEs. Two different types of seasonal influenza vaccines have been used on the market: trivalent (killed) inactivated influenza vaccine (TIV) and trivalent live attenuated influenza vaccine (LAIV). Different adverse event profiles induced by these two groups of seasonal influenza vaccines were studied based on the data drawn from the CDC Vaccine Adverse Event Report System (VAERS). Extracted from VAERS were 37,621 AE reports for four TIVs (Afluria, Fluarix, Fluvirin, and Fluzone) and 3,707 AE reports for the only LAIV (FluMist). The AE report data were analyzed by a novel combinatorial, ontology-based detection of AE method (CODAE). CODAE detects AEs using Proportional Reporting Ratio (PRR), Chi-square significance test, and base level filtration, and groups identified AEs by ontology-based hierarchical classification. In total, 48 TIV-enriched and 68 LAIV-enriched AEs were identified (PRR>2, Chi-square score >4, and the number of cases >0.2% of total reports). These AE terms were classified using the Ontology of Adverse Events (OAE), MedDRA, and SNOMED-CT. The OAE method provided better classification results than the two other methods. Thirteen out of 48 TIV-enriched AEs were related to neurological and muscular processing such as paralysis, movement disorders, and muscular weakness. In contrast, 15 out of 68 LAIV-enriched AEs were associated with inflammatory response and respiratory system disorders. There were evidences of two severe adverse events (Guillain-Barre Syndrome and paralysis) present in TIV. Although these severe adverse events were at low incidence rate, they were found to be more significantly enriched in TIV-vaccinated patients than LAIV-vaccinated patients. Therefore, our novel combinatorial bioinformatics analysis discovered that LAIV had lower chance of inducing these two severe adverse events than TIV. In addition, our meta-analysis found that all previously reported positive correlation between GBS and influenza vaccine immunization were based on trivalent influenza vaccines instead of monovalent influenza vaccines.

## Introduction

Vaccination is a highly effective and standardized public health practice; however, patients may suffer adverse events (AE) in reaction to the administered vaccine [1]. Some AEs can be serious (SAE) and even fatal. To monitor post-marketing adverse events associated with released vaccines, the Centers for Disease Control and Prevention (CDC) and the Food and Drug Administration (FDA) established the Vaccine Adverse Event Report System (VAERS) surveillance program [2]. It is important to note that adverse *event* reports in VAERS do not assert causality and, therefore, are not to be confused with adverse *effects*, which implies causal association. The primary strength of VAERS lies in the national coverage of its reporting network, so that it can pick up a rare AE incident in a timely manner. VAERS has been used in many studies that resulted in useful insights of post-vaccination incidents [3], [4], [5], [6], [7].

Since VAERS records contain high-noise data, a well-defined method is necessary to analyze VAERS entries. The VAERS reporting protocol is a *passive surveillance* system that accepts data from any reporter. Each case report is curated with individual adverse events manually assigned to the Medical Dictionary for Regulatory Activities (MedDRA) codes by VAERS personnel. However, the VAERS data entry process still introduces strong biases influenced by over- or under-reported symptoms and signs, reporters' inability to assess causality, and temporal association of the report (*e.g.*, inconclusive symptoms get inserted as post-vaccination AE). Moreover, there are no denominator data of total vaccinations for various population groups and specific vaccines; therefore, incidence rates and relative risks of specific adverse events cannot be calculated by processing the raw VAERS data [8]. However, there has been proof of plausibility of utilizing monitored post-release drug adverse event data with combinatorial bioinformatics methods in analyzing the occurrence of serious events. For example, a Bayesian network approach has been used to exploit the WHO Uppsala Center drug safety reports' pharmacovigilance database [9], [10]. The Proportional Reporting Ratio (PRR) and Chi-square significance test methods have been applied to analyze AE data in the Medicines Control Agency, and the Drug Safety Research Unit [11], [12], [13].

The MedDRA system as a coding vocabulary nomenclature has been widely used by physicians and health care researchers in annotating AE information. It has played a central role in standardizing and improving vocabulary in the scope of AE reporting. However, MedDRA has several issues in domain completeness and discrepancies with a physician's AE description that result from MedDRA's lack of a well-defined hierarchical structure [14]. A recent study of the construction of the Ontology of Adverse Events (OAE; previously known as Adverse Event Ontology (AEO) [15]) has addressed the issue of information structure of standardized vocabulary. OAE is a community-based biomedical ontology for adverse events. Biomedical ontologies are sets of terms and relations that represent entities in the real world and how they relate to each other; terms are associated with documentation and definitions, which are, ideally, expressed in formal logic to support automated reasoning [16]. OAE is now a candidate ontology in the Open Biomedical Ontology (OBO) foundry [17]. In OAE, each AE is considered as a pathological process that starts at the time of a medical intervention (*e.g.*, vaccination) and has the outcome of a symptom (*e.g.*, fever), sign (*e.g.*, increased blood glucose), or a process (*e.g.*, bacterial infection), which can be mapped to a MedDRA term. After the OAE-MedDRA term mapping, the VAERS contents transcribed with the MedDRA terminology can be analyzed by OAE that organizes adverse event terms into a logical hierarchical structure based on pathological processes of the AE symptoms.

Seasonal influenza is a common illness sufficiently fatal that the CDC Advisory Committee on Immunization Practices (ACIP) recommends that everyone 6 months of age or older should receive influenza vaccine every year (http://www.cdc.gov/mmwr/preview/mmwrhtml/rr59e0729a1.htm). Seasonal influenza is different from pandemic influenza, which only happens approximately every 40 years: 1918, 1957, 1968, and 2009. However, the risk of post-vaccination serious adverse events (SAEs), especially Guillain-Barre Syndrome (GBS), must be considered in making and implementing recommendations for use of the several types of marketed seasonal influenza vaccines. Assessing adverse events triggered by different influenza vaccines can enhance our understanding of vaccine safety.

In this study, we hypothesized that trivalent killed inactivated influenza vaccines (TIV), and intranasal spray for live attenuated influenza vaccine (LAIV), the two subtypes of trivalent seasonal influenza vaccines, induce different types of adverse events. The rationale behind managing influenza vaccines into two groups is: 1) They are two very different types of vaccines; 2) they both have been widely used after their releases, and have resulted in a significant number of adverse event records reported to VAERS; and 3) both groups of vaccines aim for protection against influenza A/B, while having different methods of administration (intramuscular injection for TIV, and intranasal spray for LAIV. Using a novel workflow of combinatorial, ontology-based detection of AE (CODAE) approach, we compared the VAERS clinic-based adverse event reports associated with these two groups of influenza vaccines. Our results have confirmed that TIV have a low signaled incidence rate of GBS and related adverse events such as paralysis and paresthesias, while recipients of trivalent LAIV have no statistically significant association with GBS or related neurological symptoms.

## Methods

### Adverse Event Data Extraction

Records of post-vaccination adverse events were queried from the CDC Vaccine Adverse Event Reporting System (VAERS; access date: May 18^th^, 2011). The query was constructed to retrieve adverse event information of killed inactivated vaccines (TIV group) consisting of *Afluria*, *Fluarix*, *Fluvirin*, and *Fluzone*, and live attenuated vaccine (LAIV group) FluMist. The names and the numbers of AEs for each type of vaccines were summarized. Some AE symptoms are common in both groups, while a significant number of symptoms are unique to each cohort. Symptoms from both AE record tables differ in rankings (number of occurrences) and the nature of adverse events themselves. We hypothesize that performing a comparative analysis of different physiological responses implied by AE symptoms associated with each vaccine could lead us to understand the underlying response mechanism of the influenza A/B vaccines.

### AE report signal detection with Proportional Reporting Ratios (PRRs)

Each group of reports (TIV and LAIV) was analyzed independently with the PRR method (as introduced by Evans et al. [11]) (Table S1). PRR calculates the proportions of specific AE(s) for a vaccine (or a group of vaccines) of interest where the comparator is all other vaccines in the VAERS database. Therefore, calculations to detect signals from the data pool utilize the total number of reports for each vaccine as a denominator to determine the proportion of all reports that fall in the type of interest (which in this case is the individual AE that was retrieved by each group of vaccine compared against reports of that particular AE in the total VAERS database pool). The PRR score of individual AEs in each group is then used as one of the composite criteria to compare for significant AEs in each group.

### Chi-square test to identify statistically significant AEs

In parallel with PRR signal detection, the Chi-square significance test for contingency tables was applied to individual AE MedDRA terms that are associated with TIV or LAIV independently [11]. The Chi-square test computes a Chi-square score and probability for each AE in each group using a 2×2 frequency/contingency table. The 2×2 contingency table was composed of four disjoint counts based on the total number of all reports in each group (37,621 TIV cases, 3,707 LAIV cases) against the overall VAERS data (616,215 cases). An AE was called significant when its Chi-square score was greater than 4, which implied P-value of approximately 0.05 or smaller [11].

### AE case report frequency as a cutoff for filtering out background noise

Besides the PRR calculation and Chi-square test, the screened PRR method (SPRR) also used a minimal sample size cutoff ([18] [19]). The original SPRR paper uses a minimal sample size cutoff of 3 case reports for each AE to be further considered. Such a constant cutoff does not work for our project since the two groups (TIV and LAIV) of case reports have different case report sizes. In our study, the sample size cutoff threshold of the number of reports for both groups was determined to be 0.2% of the total number of reports of each group. Using this cutoff, the biological implication would mean that at least 2 out of 1000 cases reported the AE of interest. The selection of the cutoff was supported by the report signal curve on the total case reports for each group (Figure S1 A and B). In either TIV or LAIV case, the 0.2% cutoff line was able to cut off many AEs which are in the bottom of the signal curve and considered as “noise”. The cutoff line is located in a similar pattern in both cases (Figure S1 A and B), suggesting that the 0.2% cutoff removes “noise” AEs in each group correspondingly. The number of cases for one AE to get called in for TIV group was evaluated to be 75 (number of reports > = 75), while the cutoff for LAIV group was evaluated at 8 (number of reports > = 8).

To determine which AEs were exclusively enriched for TIV or LAIV, we excluded AEs that appeared as common signals in both lists. We also excluded ambiguous AEs such as *no adverse event*, or those of lab test result *normal*. We were then left with 48 TIV-enriched AEs and 68 LAIV-enriched AEs. These are AEs that their corresponding PRR score is at least 2, and Chi-square is greater than 4 (approximately of probability value of 0.05 or smaller).

### Comparison of concept reorganization based on semantic similarity of the Ontology of Adverse Events (OAE)

The OAE (http://www.oae-ontology.org/) was previously named the Adverse Event Ontology (AEO) [15]. The change of the name space was applied to avoid a conflict with another ontology. OAE was downloaded from http://sourceforge.net/projects/oae/. OAE was visualized with the Protégé 4.0.2 OWL editor. For better comparison and analysis, related MedDRA terms associated with TIV and LAIV were mapped to corresponding OAE terms. However, the ontological structures of these terms in the two systems are often different. TIV- and LAIV-related AEs were classified based on the OAE structure hierarchy for comparative analyses. Specifically, the TIV- and LAIV-specific AEs and their parent term hierarchies were extracted from the OAE using the OntoFox program [16]. The hierarchical results were visualized using the Protégé-OWL editor and manually studied and compared. To compare the performances of classification using different ontologies, TIV- and LAIV-specific AE terms were also classified using SNOMET-CT, and COSTART/MedDRA.

## Results

### Overall study design

Data obtained by clinical observations are often high in statistical noise, which sometimes leads to temporal associations that are wrongly believed to be causal [20]. In this study, we demonstrated that, by combining multiple statistical and bioinformatics methods, background noise and irrelevant information can be reduced to a minimal level. This also allows a meaningful interpretation of data. Scientists can draw a sensible hypothesis from these processed data.

**Figure 1:**
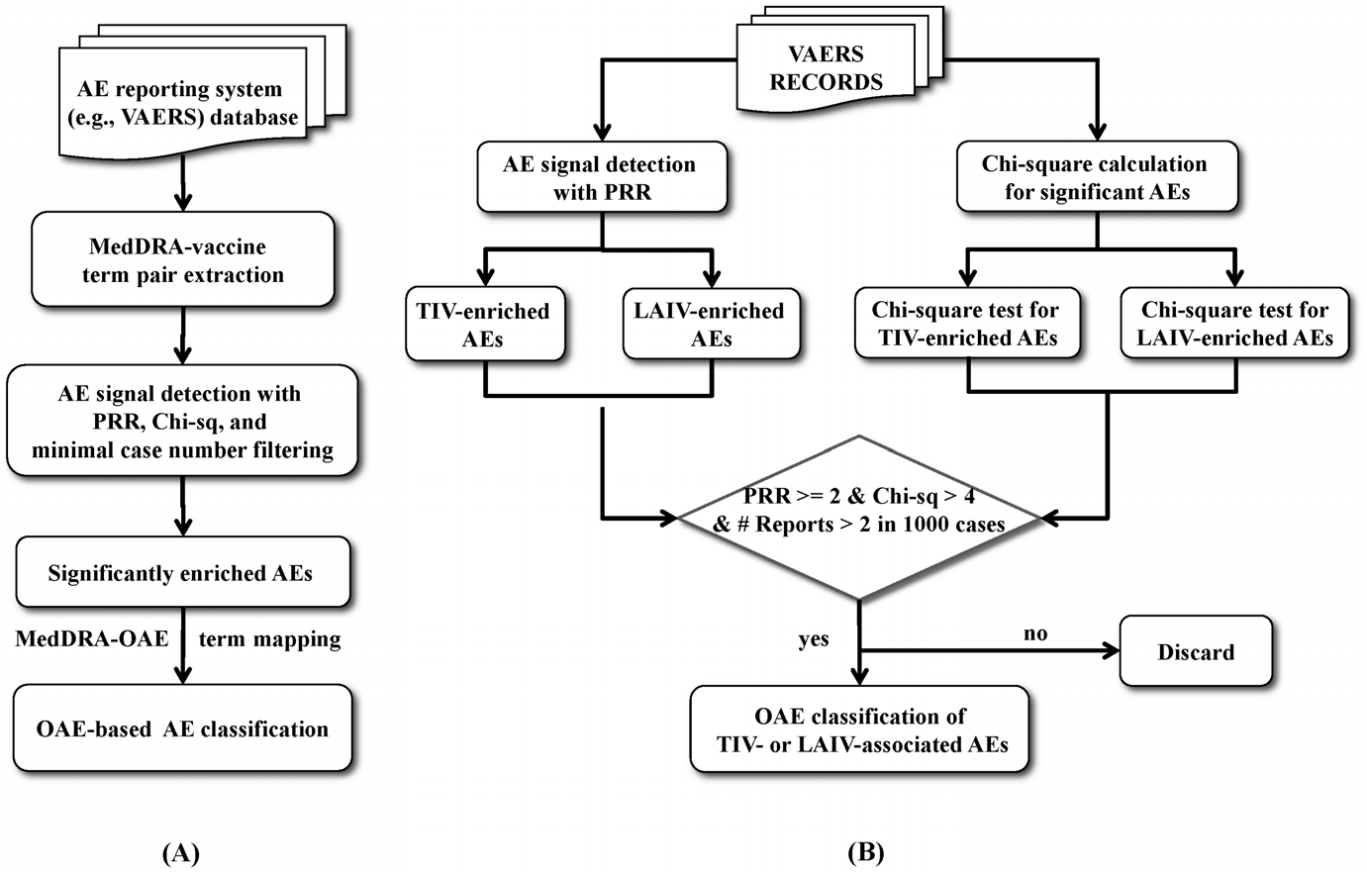
Workflow of the CODAE integrative AE bioinformatics analysis. A generalized version of CODAE for detection of significant AE terms for one vaccine or one group of vaccines is outlined in (A). See details in the text. An expanded CODAE solution to analyze and compare AEs associated with the two vaccination groups is shown in (B). VAERS records were retrieved based on the query criteria of 4 TIVs (Afluria, Fluarix, Fluvirin, and Fluzone) year 1990–2011 and 1 LAIV (FluMist) year 2003–2011. Parallel analyses of the Proportional Reporting Ratios and Chi-square significant test were performed on individual AEs to identify enriched and significant AEs in each group. Base level filtration of 0.2% of total number of reports was also applied to each AEs. AEs that were identified to have PRR > = 2, Chi-square > = 4, and number of reports > = 0.2% of total reports were then classified based on OAE hierarchical structure. Classification of AEs filtered out AEs that overlapped between the 2 groups.

Our Combinatorial Ontology-based Detection of vaccine Adverse Events method (CODAE) is outlined in Figure 1. The generalized version of CODAE is for detection of significant AE terms for one vaccine or one group of vaccines (Figure 1A). As the first step of CODAE analysis, the information of vaccine-MedDRA term associations is extracted from a spontaneous AE reporting system (*e.g.*, VAERS). Then a reliable and robust bioinformatics method, such as the commonly used Screened Proportional Reporting Ratio (SPRR) [11], [18], can be used to identify statistically sound AE signals. Then the identified AEs can be classified using an ontology-based classification method.

The screened PRR (SPRR) methodology is typically used in detection of vaccine adverse events (VAEs) from spontaneously reported data. PRR is a component of SPRR for VAE signal detection [11]. However, an association between a vaccine and VAE from PRR calculation might not be truly statistically significant due to the lack of well-defined null distribution [18]. To overcome this concern, SPRR methodology includes an additional Yates-corrected Chi-square score calculated to detect the significance level of each AE term in the specific group of vaccines. A Chi-square score of 3.84 equals to a P-value of 0.05. Therefore, a Chi-square score of greater than 4 is approximately equivalent to a P-value of less than 0.05. To ensure the specificity of the study, SPRR also includes a minimum number of reports as a filter to eliminate random occurrences. In summary, SPRR uses the following screening criteria; PRR > = 2, Yates-corrected Chi-square > = 4, and minimal case report number > = 3 [11].

In our CODAE strategy, we modified the SPRR methodology [11], [18] with an adjustable cutoff for filtering AEs which have a low number of case reports. In SPRR and previous smaller PRR-based studies, this cutoff number was recommended to be at least 3 occurrences [11], [21]. Since TIV and LAIV are each associated with a large number of case reports, at a cutoff number of VAERS reports = 3, the significant AE lists associated with TIV or LAIV cover hundreds of AEs. Moreover, the case-report populations vaccinated by TIV and LAIV differ by size. Since one major objective of our study is to compare the AEs and SAEs independently induced by TIV and LAIV, we determined that the number of reports per AE must be at least >0.2% of total reports, *i.e.*, at least 75 (out of total 37,621) for TIV and 8 (out of total 3,707) for LAIV. The necessity and accuracy of this setting are reflected by the large numbers of AEs having low AE frequencies below the 0.2% cutoff shown in Figure S1. The 0.2% cutoff provided us better manageability of the sets of AEs studied in each cohort.

This generalized CODAE workflow (Figure 1A) can be expanded to analyze and compare AEs associated with two groups of vaccines such as TIV and LAIV (Figure 1B). In our TIV and LAIV VAE study, the Chi-square analysis and PRR of each group were performed separately in parallel with each other. Those AEs with PRR score greater than 2 and Chi-square score greater than 4 were kept for further studies. These AEs are statistically significant, TIV- or LAIV-enriched AEs.

After significant AE signals are detected, we then classified the signals using an ontology-based method. Each AE signal in VAERS is represented by a MedDRA term with a unique identifier. All these MedDRA terms were mapped to specific terms of the Ontology of Adverse Events (OAE). The OAE hierarchy was used to classify the AE signals.

### Overall results: extracting differential AE profiles from VAERS TIV and LAIV data

As of May 18, 2011, there were 7,520 MedDRa AE terms and 616,215 VAERS case records (one record may contain multiple AEs) listed for 75 vaccines reported to VAERS. The two subsets of seasonal influenza vaccines studied (TIV and LAIV) held 3,582 AE terms in total, and the comparison set contained 37,621 TIV reports and 3,707 LAIV reports. Following the overall analysis pipeline (Figure 1), TIV- or LAIV- enriched AEs were determined by Chi-square score (>4), PRR score (>2), and the number of reports (0.2% of total reported cases, *i.e.*, > = 75 for TIV and > = 8 for LAIV).

**Figure 2:**
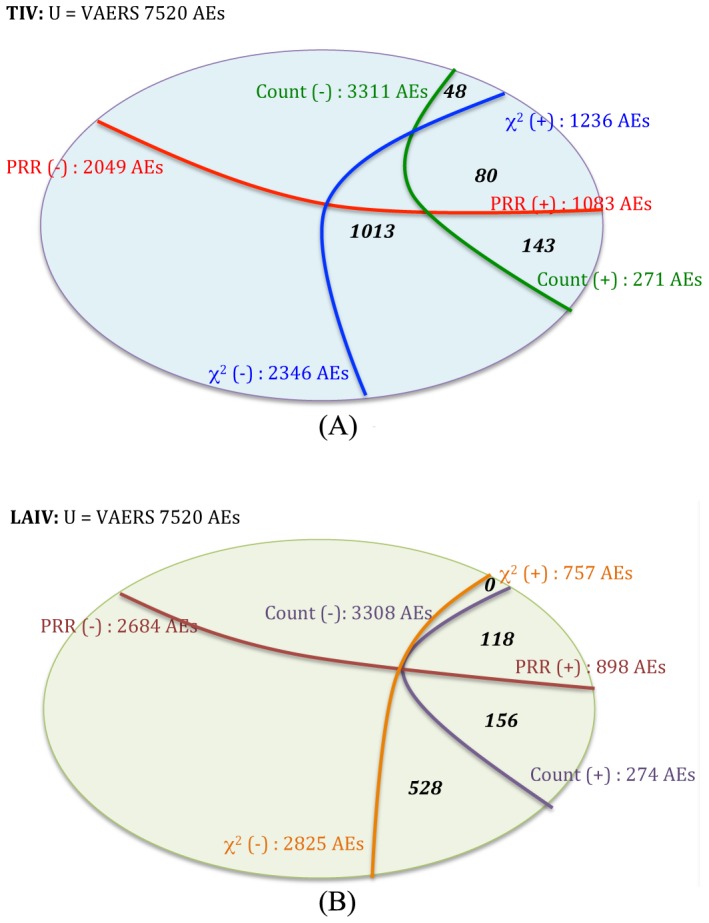
Venn diagram summary of the three filtering criteria in each group of vaccines from the pool of 3,582 AEs analyzed in TIV and LAIV and the universe of 7,520 AEs in the entire VAERS database. Chi-square value of > = 4 – χ^2^(+), or <4 – χ^2^(−); PRR > = 2 – PRR(+), or PRR <2 – PRR(−); and number of reports >0.2% of total reported cases (*i.e.*, > = 75 in TIV or > = 8 in LAIV) – count(+), or else – count(−).

Figure 2 provides a Venn diagram showing the results after the three criteria of AE selection were applied in this study. In the TIV group, 1,236 AEs have their Chi-square scores greater than 4 (labeled as χ^2^(+)), while 2,346 AEs did not pass this condition (labeled as χ^2^(−)). In terms of PRR analysis, 1083 AEs contained a PRR score of at least 2 (labeled as PRR(+)), while 2,499 AEs did not pass the PRR criterion. In total, 271 AEs passed the condition of sample size of at least 75 (labeled as count(+)), while 3,311 AEs did not (labeled as count(−)). These numbers indicate that, even though sample size filtering screened out the majority of the low-signal AEs, additional filtering by χ^2^ and PRR scoring provided screening measures that could help detect true signals of enriched AEs with high significance. Among 271 AEs that passed the sample size screening, there existed 223 AEs that passed the χ^2^ test and 128 AEs that passed PRR evaluation. There were 80 AEs that overlapped within the screening of χ^2^ and PRR tests, leaving 48 AEs out as the result of 3-criteria elimination.

In the LAIV group, 757 AEs passed the χ^2^(+) filtering, 2,825 remained in (χ^2^(−)). Based on frequency filtering, for the PRR analysis, 898 AEs passed PRR condition (PRR(+)) while 2,684 AEs were excluded by PRR (PRR(−)). 274 AEs contained at least 8 records per AE (count(+)), and 3,308 were left in count(−) group. Note that the LAIV cohort had identical screening results with χ^2^ and PRR after the sample size cutoff. This phenomenon was considered coincidental and did not occur in the TIV case, and should not suggest that either screening method did not deliver further or useful filtering.

**Figure 3:**
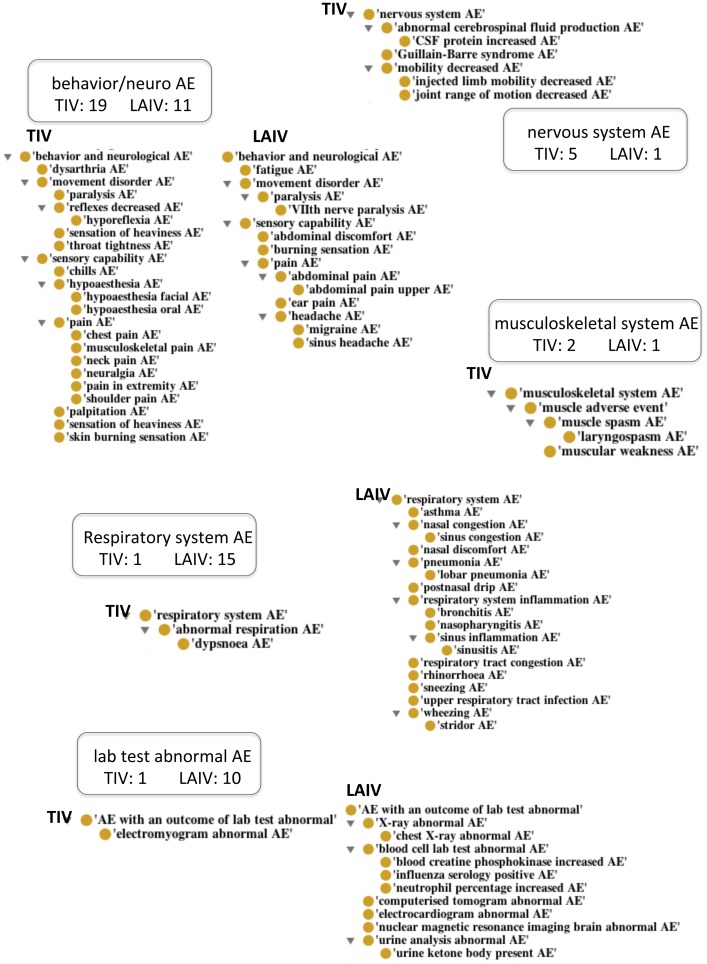
Diagram of AE counts grouped by related symptoms. Behavior/neurological system contains the most adverse events distributed in two groups of vaccines (40 adverse events; 25 in TIV, 15 in LAIV) but the clusters are significantly different in processes. TIV's behavior/neurological AEs are much more closely related to those of muscle and movement disorder while LAIV's behavior/neurological AEs cluster around pain in the head. Respiratory system AEs is listed as the most significant cluster in LAIV group with 16 AEs. Full listing can be found in Table 1 (TIV) and Table 2 (LAIV).

There were 80 TIV AEs and 118 LAIV AEs that passed all three conditions with 31 AEs overlapping between the two lists. After screening out ambiguous or common AE terms, 48 AEs were included in the TIV analysis, and 68 AEs were included in the LAIV analysis. Tables 1 and 2 summarize the lists of AEs for TIV and LAIV that were used for analytical clustering, respectively. The AEs in these two tables were classified based on an ontological method using the Ontology of Adverse Events (OAE) (Figure S2). For comparisons, the classifications of TIV- and LAIV-enriched AE terms were also generated using MedDRA (Figure S3) and SNOMED-CT (Figure S4). The enriched AE terms represent those AE terms that were statistically “enriched” in one group compared to the other group (P-value <0.05). The comparison of the results from these classifications is described in the Discussion section. Of the three classification systems, the OAE classification method performed the best for the purpose of classification. This was considered based on feasibilities of structural organization of the ontology, clarification and definition of terms, and domain coverage (see detailed discussion in the Discussion section). The most differential clusters of the adverse events induced by these two types of influenza vaccines are summarized in Figure 3 and explained below in detail.

**Table 1:** TIV-specific adverse events. 37,621 TIV-induced AE cases were reported.

Adverse Event	Count	PRR(TIV)	Chi-sq (TIV)
**AE with an outcome of lab test abnormal**			
Electromyogram abnormal	107	4.87	248.14
**behavior and neurological AE**			
Dysarthria	91	2.80	75.22
**behavior and neurological AE -> movement disorder AE**			
Paralysis *	181	2.22	105.00
Hyporeflexia (r)	77	2.46	56.62
**behavior and neurological AE -> sensory capability AE**			
Pain	4516	2.12	2475.50
Chills	2286	2.78	2237.97
Pain in extremity	2106	3.40	2944.16
Paraesthesia (r)	1360	2.29	858.59
Hypoaesthesia (r)	1035	2.94	1114.16
Chest pain	725	2.37	492.53
Neck pain	543	2.06	260.28
Throat tightness	449	5.20	1134.22
Musculoskeletal pain (r)	432	4.00	767.24
Palpitations	267	2.70	240.23
Feeling cold	186	2.65	161.93
Skin burning sensation	113	3.04	127.95
Sensation of heaviness	100	2.99	109.56
Shoulder pain	78	3.40	107.20
Neuralgia (r)	77	2.37	52.32
**cardiovascular disorder AE**			
Heart rate increased	397	2.47	297.95
Hypertension	306	2.28	189.79
Blood pressure increased	216	4.11	398.44
Injection site haematoma	175	4.08	318.99
**digestive system AE**			
Dysphagia	299	2.23	175.09
Dry mouth	75	2.68	66.49
**eye disorder AE**			
Eye discharge	135	6.05	406.32
Eye irritation	115	4.62	249.17
**hometostasis AE**			
Pharyngeal edema	256	4.30	503.52
Swollen tongue	158	4.56	336.46
Tongue edema	105	2.51	81.09
Local swelling	88	2.05	40.97
**medical intervention** (not under ‘adverse event’)			
Accidental overdose	83	4.59	177.98
**muscle adverse event**			
Muscular weakness (r)	594	2.89	614.83
**musculoskeletal system AE**			
Laryngospasm	143	2.83	141.06
**nervous system AE**			
Guillain-Barre syndrome *	606	4.63	1321.69
Mobility decreased (r)	161	3.40	221.81
**nervous system AE -> mobility decreased AE**			
Injected limb mobility decreased (r)	561	4.72	1253.55
Joint range of motion decreased (r)	317	4.36	635.69
**respiratory system AE**			
Dyspnea	2088	2.18	1180.96
**skin adverse event**			
Flushing	403	3.00	447.05
Eye pruritus	168	9.06	754.39
Hot flush	109	3.37	147.90

Note:

* = serious adverse event,

(r) = related to serious adverse event.

**Table 2:** LAIV-specific adverse events. 3,707 TIV-induced AE cases were reported.

Adverse Event	Count	PRR(LAIV)	Chi-sq(LAIV)
**AE with an outcome of lab test abnormal**			
Influenza serology positive	32	28.46	715.61
Chest X-ray abnormal	24	4.00	51.89
Blood creatine phosphokinase increased	19	3.96	40.33
Blood glucose increased	18	2.25	11.95
Urine analysis abnormal	15	2.78	16.43
Computerised tomogram abnormal	13	2.23	8.44
Nuclear magnetic resonance imaging brain abnormal	13	2.74	13.77
Electrocardiogram abnormal	11	2.36	8.27
Neutrophil percentage increased	11	3.00	14.13
Urine ketone body present	10	7.11	49.64
Lymphocyte percentage decreased	9	3.13	12.51
**behavior and neurological AE**			
Headache	383	2.22	257.06
Fatigue	159	2.16	97.17
Abdominal pain upper	54	3.49	92.50
Ear pain	22	3.01	28.48
Migraine	21	2.54	18.85
Abdominal discomfort	15	3.38	24.20
Burning sensation	15	2.25	9.97
Sinus headache	14	18.72	208.53
VIIth nerve paralysis	11	11.08	93.29
Facial paresis	9	5.37	30.53
Ataxia	8	3.50	13.72
**cardiovascular disorder AE**			
Epistaxis	71	14.71	823.81
Pericarditis	9	3.97	19.16
**digestive system AE**			
Retching	12	3.27	18.19
Dry throat	10	10.32	78.17
**gustatory system AE**			
Throat irritation	17	3.05	22.55
**errored drug administration**			
Expired drug administered	503	90.04	28507.94
Inappropriate schedule of drug administration	169	4.15	390.47
**eye disorder AE**			
Photophobia	17	2.66	16.90
Eye irritation	10	3.41	16.37
Visual impairment	9	3.05	11.92
**homeostasis AE**			
Swelling face	44	2.51	38.58
Eyelid edema	13	2.03	6.49
**immune system disorder**			
Immunization reaction	9	2.43	7.30
**infection adverse event**			
Croup infectious	11	9.58	78.80
**injury and procedural complication AE**			
Pregnancy test positive	10	3.21	14.60
**medical intervention**			
Drug exposure during pregnancy	77	3.90	159.92
Accidental exposure	30	20.56	490.83
Vaccination error	13	30.19	306.90
Underdose	11	11.64	98.65
Drug administration error	9	5.81	34.07
**muscle disorder AE**			
Bronchospasm	8	4.60	21.52
**respiratory system AE**			
Rhinorrhea	210	9.47	1493.03
Nasal congestion	177	11.64	1593.58
Sneezing	53	10.78	436.15
Pneumonia	43	2.01	20.90
Sinusitis	43	5.92	167.24
Asthma	35	2.07	18.45
Respiratory tract congestion	32	7.68	175.14
Upper respiratory tract infection	25	2.22	16.05
Nasopharyngitis	25	4.08	55.69
Bronchitis	23	2.81	25.84
Sinus congestion	13	8.49	80.57
Nasal discomfort	12	47.77	422.18
Stridor	10	3.82	19.93
Postnasal drip	10	20.90	166.27
Lobar pneumonia	10	15.77	124.77
**skin adverse event**			
Pruritus generalized	15	2.94	18.45
Rash pustular	15	2.19	9.29
Henoch-Schonlein purpura	15	8.20	89.03
**social behavior AE**			
Activities of daily living impaired	23	2.36	17.29
Impaired work ability	8	3.96	16.94

### Distinctive underlying biological activities were associated with the two groups of influenza vaccines

In summary, biological systems highlighted in TIV AEs were the behavior/neurological system, immune system, and muscle/nervous systems. LAIV AEs appeared to cluster heavily in the respiratory system. Behavior/neurological adverse events were triggered by both TIV and LAIV. However, manual examination revealed that TIV-induced behavior/neurological adverse events clustered around muscular, motor and movement disorders, and LAIV-induced adverse events were mainly pain symptoms in the head. Figure 3 illustrates the reorganization of TIV- and LAIV-induced AEs in detail based on the Ontology of Adverse Events [15]).

The TIV-associated AEs clustered and enriched in movement and muscle disorders included joint range of motion decreased, mobility decreased, muscular weakness, Guillain-Barré syndrome (GBS), paralysis, and hyporeflexia. Another set of TIV-induced adverse events that was observed exclusively in TIV was edema (homeostasis and fluid dysregulation) in various parts of the body. Detailed description of GBS as a TIV-enriched severe AE is provided in the following section. No inflammatory responses came up as significant TIV-induced AEs (as opposed to LAIV).

LAIV influenza vaccine triggered other sets of biological activities in processes of the respiratory system (*e.g.*, sinus headache, nasal congestion) and respiratory system disorders that were characterized by inflammation – upper respiratory tract infection, pneumonia, bronchitis, and nasopharyngitis. These AEs may be associated directly with the intranasal mode of administration of the LAIV. Fifteen distinct adverse events were reported as over-represented respiratory system disorders. Activities in the hematopoietic system also suggested evidence in responses to stimulus related to inflammation. Furthermore, LAIV-induced adverse events showed a set of activities involved in the gustatory system and gustatory system-related activities.

### Severe AEs are highly enriched in killed-inactivated influenza vaccine group

Based on FDA's definition of serious adverse event (SAE) (http://www.fda.gov/safety/medwatch/howtoreport/ucm053087.htm), SAE is an adverse event that results in serious or fatal health condition such as death, permanent damage, or hospitalization. It should be noted that 2 TIV-specific AEs were SAEs. These included GBS (Chi-square: 1172.79/P-value: 4.99E-257, PRR score: 4.63), and paralysis (Chi-square: 85.48/P-value: 2.34E-20, PRR score: 2.22). AEs that are related to these SAEs and also enriched in TIV are hypoaesthesia, mobility decreased, joint range of motion decreased, musculoskeletal pain, paraesthesia, and neuralgia.

The association of GBS with influenza vaccination has long been debated. GBS is a serious immune system disease that has been reported repeatedly as a rare complication after influenza vaccine immunization [19], [22]. GBS is categorized under immune system disorder based on cause of disease, or nervous system disorder based on its biological responses (muscular weakness, and paralysis). It is notable that a significant number of AE cases reported as a consequence of administering influenza TIV vaccines are related to loss of muscle strength in various forms without the development of GBS. GBS often results in a key symptom of movement disorder. There are reports of associations between GBS and TIV influenza vaccines, but the cause-effect relations remain inconclusive (Table S1).

Another evidence for TIV-associated compromised muscular system activities was the significantly ranked abnormal electromyogram result from TIV AE case reports. Electromyogram (EMG) is a test that evaluates electrical activity of muscle. Often, physicians utilize EMG as a method to diagnose GBS and other muscle-related disorders [23], [24]. Observation of both abnormal lab test result AE (electromyogram abnormal) and physiological evidence in nervous and muscular disorders pointed toward TIV-triggered inter-connecting activities in the human body that were key symptoms of severe AEs (GBS and paralysis).

No SAEs were enriched among the LAIV AEs listed in Table 2. Pain in the head/neck area exists exclusively on the LAIV list, which may be explained by the route and method of LAIV administration (nasal spray). These respiratory system disorder AEs, for example, include pneumonia (Chi-square: 20.9/P-Value: 4.85E-06, PRR score: 2.01), lobar pneumonia (Chi-square: 124.77/P-value: 5.7E-29, PRR score: 15.77), bronchitis (Chi-square: 25.84/P-value: 3.71E-07, PRR score: 2.81), and upper respiratory tract infection (Chi-square: 16.05/P-value: 6.16E-05, PRR score: 2.22). Operational errors such as expired drug administered, or inappropriate schedule of drug administration reported as post-vaccination AE were reported with higher significance in LAIV.

### Post-immunization Guillain-Barré Syndrome (GBS) in TIV recipients occurred at a higher rate per number of reports than in LAIV

**Figure 4:**
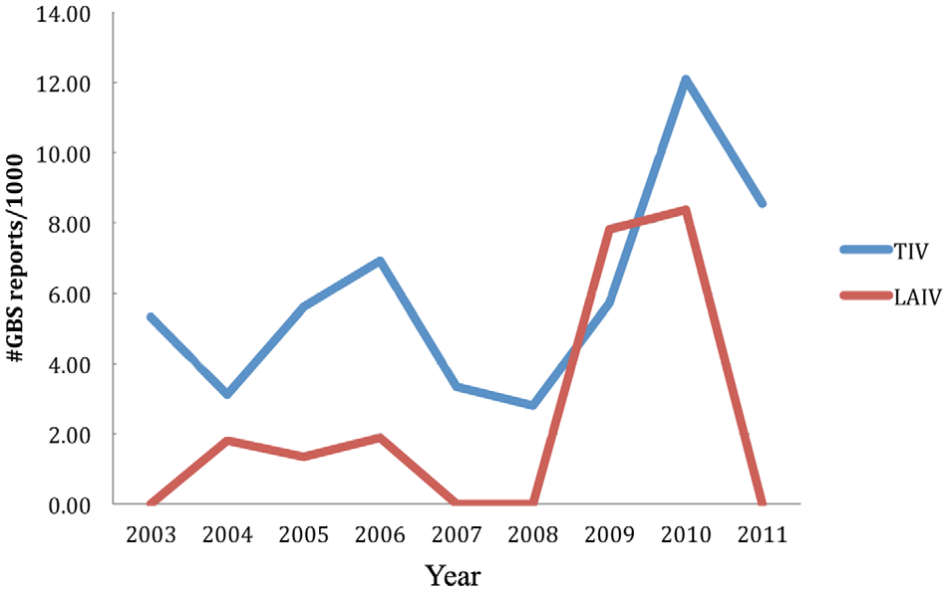
Comparison of reporting rates of GBS cases associated with TIV and LAIV administrations. The Y-axis is the number of GBS cases per 1000 case reports for either TIV or LAIV group. The comparison starts the year when both groups have available data in VAERS.

Although enriched in TIV data, GBS is quite an infrequent incident. Based on our literature review, the incidence rate of GBS in influenza vaccine recipients is considered rare, approximately 1 in 100,000 [22]. Reports of GBS in LAIV seem to be even rarer. Post-vaccination GBS was ranked among the over-represented AEs in all scoring matrices of TIV group (Table 1). In contrast, the information retrieved via this study showed a small reporting rate of GBS in the LAIV group that the statistical analysis did not recognize GBS as LAIV-enriched in Table 2 (ranging from 0–8.37 per 1,000 cases in LAIV in comparison to 2.81–12.1 per 1,000 cases in TIV, data from 2003–2010). However, without consideration of PRR, GBS would pass the criteria of Chi-square and number of reports in both TIV and LAIV. Manual examination and cross referencing of input data (VAERS records) confirmed that the incidence was not manufacturer lot specific. Investigation of the numbers of GBS cases reported per year in each group suggested that LAIV was less likely to be associated with post-immunization GBS than TIV (Figure 4).

**Figure 5:**
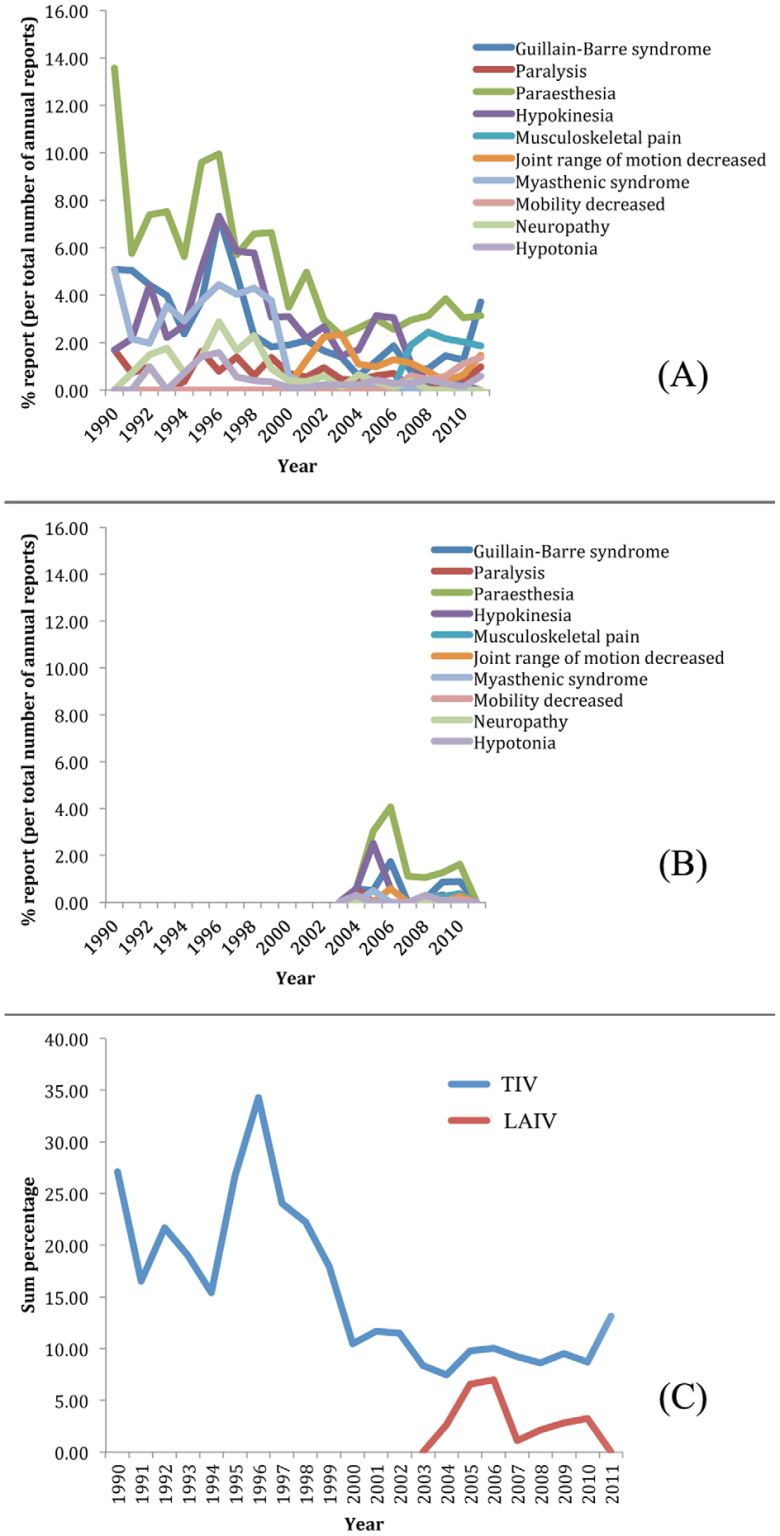
Comparison of yearly report distributions of GBS and GBS-related adverse events associated with TIV and LAIV. LAIV was recently released and therefore data available are from 2003 onward. The raw number of occurrences was scaled to percentages by the number of reports in each year. The percentages of yearly case reports of GBS and other GBS-related symptoms associated with TIV and LAIV are displayed in (A) and (B), respectively. The combined percentages of GBS and related AEs associated with TIV and LAIV are depicted in (C).

To further investigate the reporting rate of GBS and GBS-related AEs among the patients who reacted to the trivalent seasonal influenza vaccines (both TIV and LAIV), we first tried to determine whether or not these reported incidences were specific to the year of reports. Figures 5A and 5B display the percentage ratio of the reported cases of TIV- and LAIV-induced GBS and other related symptoms per calendar year of reports. These AEs include symptoms resulting in movement and muscular disorders: paralysis, paraesthesia, hypokinesia, musculoskeletal pain, joint range of motion decreased, myasthenic syndrome, mobility decreased, neuropathy, and hypotonia. We extended the scope of GBS examination to include other muscular and nervous disorders in an attempt to avoid the possibility of overlooking AEs that were closely related to GBS. These cases would have been ignored as non-important when focusing on GBS alone. Figure 5C shows the combined percentage of these selected AEs as one cluster based on year of reports. The denominator for the individual year calculation is the number of cases reported in that particular year. Figures 5 (A–C) have pointed towards the over-represented incidence rate of GBS and GBS-related AEs in TIV.

**Figure 6:**
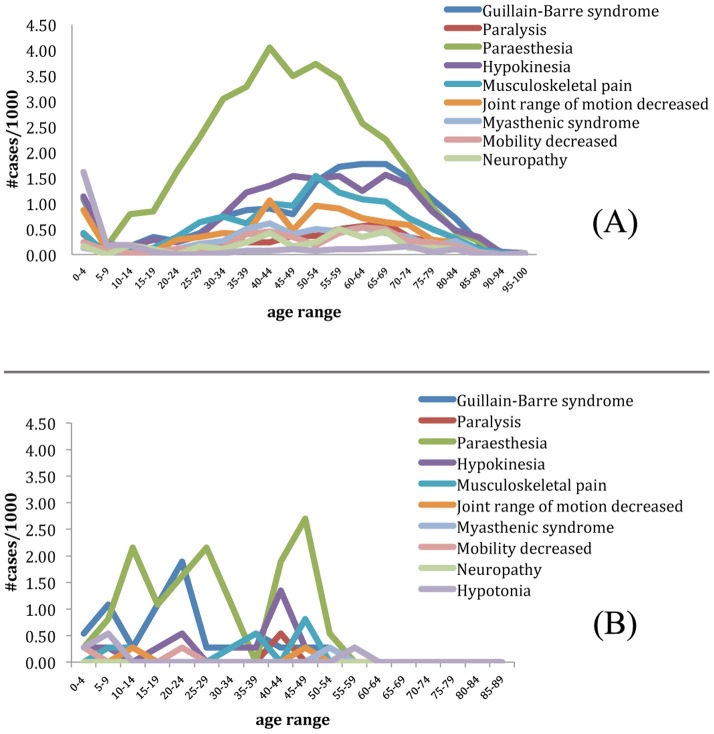
Comparison of case report distributions of GBS and GBS-related adverse events associated with TIV and LAIV based on age using the data from 2003. (A) In TIV, all but one selected AEs (paraesthesia) followed the expected age range of the populations who were more at risk of GBS (young children and the elderly). (B) In LAIV recipients, age distribution is scattered across all age ranges.

Since LAIV surveillance data became available only in late 2003 after FluMist was released, the comparison of TIV and LAIV by Chi-square significance test against each other could only be calculated from 2004 on. Table 3 summarizes TIV's probability value of how much more GBS and GBS-related AEs are represented in comparison to LAIV. The results show that all but three P-values are smaller than 0.05, signifying that GBS and GBS-related AEs are more enriched in TIV. One of those three years is a statistical artifact since the raw number of occurrences in LAIV is equal to 0 (partial year 2003). Figures 6A and 6B plot the age-range percentage ratio in which each examined AE occurred based on the total sample size in this study of TIV and LAIV, respectively. The results indicate that all GBS and GBS-related AEs occurred at a higher rate in the early age group (0–5 years) with another trend of increasing occurrence in middle to later age range (40–75) (Figure 5A). There was no suggestion of age pattern in GBS and GBS-related AE occurrences in the LAIV group (Figure 5B).

**Table 3:** Summary of statistical analysis testing if GBS and GBS-related AEs occur independently of vaccine type.

year	#GBS and GBS-related AEs(TIV)	total cases reported in that year (TIV)	# GBS and GBS-related AEs(LAIV)	total cases reported in that year (LAIV)	P-Val (selected AEs occur independently of TIV)
2003	150	1790	0	34	7.81E-02
2004	163	2187	9	339	1.10E-03
2005	278	2842	13	198	1.37E-01
2006	244	2430	12	172	1.92E-01
2007	292	3165	2	180	1.84E-04
2008	321	3725	14	660	7.01E-09
2009	498	5231	36	1272	6.46E-15
2010	625	7165	26	797	9.44E-08
2011	134	1021	0	75	8.12E-04

Note: GBS-related muscle and neurological AEs studied here included musculoskeletal pain, paraesthesia, and muscular weakness.

Age range distribution of influenza vaccine-associated GBS and other related symptoms is depicted in Figure 6. Figure 6A focuses on TIV recipients. All but one selected AEs (paraesthesia) followed the expected age range of the populations who were more at risk of GBS (young children and the elderly). Figure 6B describes the age distribution of LAIV recipients who developed the same symptom AEs as those in Figure 6A. Unlike TIV recipients, the age distribution of LAIV recipients is scattered across all age ranges. The comparison of the age distributions in these two groups indicates that TIV (but not LAIV)-associated GBS occurrence follows a similar trend of GBS occurrence in the influenza vaccine recipients who are young children and elderly [25], [26], [27], [28], while LAIV-associated GBS cases occur in all age ranges as observed in general cases [29].

### Meta-analysis of influenza vaccine AE reports suggests association between GBS and TIV (but not MIV)

Our meta-analysis of influenza vaccine AE reports suggests that monovalent inactivated influenza vaccines are associated with fewer reports of SAE compared to trivalent inactivated influenza vaccine (Table 4). The majority of peer-reviewed publications on influenza vaccine-associated GBS in recent years were studies of monovalent influenza vaccines. Of 19 influenza vaccine-related GBS from our literature survey (Table 4), five concluded elevated incidence rate or reporting ratios or GBS as the most common reported SAE, four described inconclusive correlation of GBS to influenza vaccination, and 10 stated that there were no positive correlations of GBS to influenza vaccine immunization. The studies in this literature survey were performed on the set of both trivalent and monovalent influenza vaccines. All five studies indicating that GBS had a positive correlation with influenza vaccine immunization were based on trivalent influenza vaccines.

**Table 4:** Summary of association of GBS to influenza vaccines in peer-reviewed literature.

Author	Title	Publication year	Type(s) of Influenza vaccine(s) studied	DB used	Method	Conclusion
Baxter, R. [43]	Recurrent Guillain-Barre Syndrome Following Vaccination	2012	TIV	Kaiser Permanente Northern California	review of medical records of GBS confirmed cases	low risk of recurrent GBS
Lee, S.J. [44]	Neurologic adverse events following influenza A (H1N1) vaccinations in children	2012	H1N1 MIV	N/A	single case study of 14 cases Nov.09–Mar.10	No major Neurologic AEs
Andrews, N. [45]	Guillain-Barre syndrome and H1N1 (2009) pandemic influenza vaccination using an AS03 adjuvanted vaccine in the United Kingdom: Self-controlled case series	2011	H1N1 MIV (2009)	N/A	review of patient records of post-vaccination GBS cases using self-controlled case series method on case identified in hospital episode statistics	no evidence of increased risk of GBS 6 weeks after vaccination
Cheo, Y.J. [36]	Serious adverse events follwing receipt of trivalent inactivated influenza vaccine in Korea 2003–2010	2011	TIV	Korea National Vaccine Injury Compensation Program (2003–2010)	retrospective review of clinical records, case investigation reports, conference materials and billing records.	GBS was the most-common SAEs reported after TIV immunization
Dieleman, J. [46]	Guillain-Barre syndrome and adjuvanted pandemic influenza A (H1H1) 2009 vaccine: multinational case-control study in Europe	2011	H1N1 MIV (2009)	N/A	GBS and Fisher syndrome case-control study (matched on age, sex, index date, and country)	no increased risk of GBS
Lee. G.M. [22]	H1N1 and Seasonal Influenza Vaccine Safety in the Vaccine Safety Datalink Project	2011	H1N1 MIV, LAMV, TIV, LAIV	Vaccine Safety Datalink	Nov'09–Apr'10 weekly signal detection analysis using self-controlled design /or/ current-vs-historical comparison	No association of GBS and other neurologic outcomes. For MIV - signal of Bell's Palsy. Higher reports of GBS in TIV than LAIV.
Sejvar, J.J. [47]	Guillain-Barre Syndrome Following Influenza Vaccination: Causal or Coincidental?	2011	MIV, TIV	N/A	analysis of past studies	question not answered, causality of GBS with regards to influenza vaccination remained inconclusive
Verity, C. [48]	Guillain-Barre syndrome and H1N1 influenza vaccine in UK children	2011	H1N1 MIV (?)	N/A	Sep'09–Aug'10 follow-up clinical questionairs	no association of GBS to influenza vaccination
Williams, S. E. [49]	Causality assessment of serious neurologic adverse events following 2009 H1N1 vaccination	2011	H1N1 MIV (2009)	VAERS	review of SAE reports in Oct'09–Mar'10	inconclusive association assessment, investigation of GBS and other SNAEs causality is difficult, VAERS reporting process can be improved
Burwen, D.R. [37]	Evaluation of Guillain-Barre Syndrome among recipients of influenza vaccine in 2000 and 2001	2010	not specified	Medicare claim data & hospital records	Incidence Rate Ratio	slightly non-significant elevated incidence rate ratio of GBS for all seasons combined
McNeil, M.M. [50]	A cluster of nonspecific adverse events in a military reserve unit following pandemic influenza A (H1N1) 2009 vaccination-Possible stimulate reporting?	2010	H1N1 MIV	VAERS	surey & review of index cases' VAERS reports, hospital records, vaccination status, aiagnostic results and outcome in comparison to VAERS reports of the same screen lot	GBS in the index case not confirmed, possbile stimulated reporting among reporters from the index case's cohort
Vellozzi, C. [51]	Adverse events following infuenza A (H1N1) 2009 monovalent vaccines reported to the Vaccine Adverse Event Reporting System, United States, October 1, 2009–January 31, 2010	2010	H1N1 MIV	VAERS	empirical Bayesian data mining and reporting proportions with clinical review of reports	death, GBS, and anaphylaxis were rare (<2 per million doses from ∼10,000 VEARS reports)
Evans, D. [52]	“Prepandemic” Immunization for Novel Influenza Viruses, “Swine Flu” Vaccine, Guillain-Barre Syndrome, and the Detection of Rare Severe Adverse Events	2009	clinical trials of H5N1	N/A	review of past pandemic influenza studies	GBS association with Influenza vaccination remained inconclusive. Possible mechanism of GBS association with influenza vaccine was discussed.
Vellozzi, C. [38]	Safety of trivalent inactivated influenza vaccines in adults: Background for pandemic influenza vaccine safety monitoring	2009	TIV (1990–2005)	VAERS	PRR, review of reports of recurrent events and death	slightly elevated risk of SAEs, GBS - most frequently reported SAE, GBS requires continued monitoring
Juurlink, D.N. [39]	Guillain-Barre Syndrome after influenza vaccination in adults: a population-based study	2006	not specified	N/A	self-matched case-series method and time-series analysis	Influenza vaccination is associated with increased risk of hospitalization due to GBS
Izurieta, H.S. [53]	Adverse Events Reported Following Live Cold-Adapted, Intranasal Influenza Vaccine	2005	LAIV	VAERS (2003–2005)	report rate per 100,000 vaccinees	No unexpected serious risks, no GBS, may rarely cause anaphylaxis
Kao, C. [54]	Guillain-Barre syndrome coexisting with pericarditis or nephrotic syndrome after influenza vaccination	2004	not specified	N/A	2 individual case studies	pericarditis or onset nephrotic syndrome may coincidoe with GBS development.
Geier, M.R. [40]	Influenza vaccination and Guillain Barre syndrome	2003	not specified 1991–1999	VAERS	stat. analysis with Corel's Quattro Pro	increased risk of acute GBS and severe GBS in comparison to tetanus-diphtheria control group
Lasky, T. [41]	The Guillain-Barre Syndrome and the 1992–1993 and 1993–1994 influenza vaccines	1998	not specified	VAERS	patient survey and review of hospital discharging records based on VAERS reports	elevated relative risk of GBS 6 weeks after vaccination when combined the two reporting years. No increase in the risk when looking at each year individually.

Abbreviations: MIV – Monovalent Inactivated Influenza Vaccine, TIV – Trivalent Inactivated Influenza Vaccine, LAIV – Live Attenuated Influenza Vaccine, VAERS – Vaccince Adverse Event Reporting System.

## Discussion

To study vaccine AEs associated with a specific vaccine, two types of immunization population denominators can be used. One is the total number of people immunized for one single vaccine in a region during a given period. Another type of population denominator is the total number of people who reported AE cases to VAERS for all vaccines in a region during a given period. Like other VAERS bioinformatics studies, our approach uses the second immunization population denominator [18]. Although the VAERS spontaneous reporting system lacks a true control (i.e., people randomized to receive a placebo), our bioinformatics method analyzed AEs associated with a vaccine using all other vaccines as a quasi-control group for comparison [18].

In our study, we did not directly compare TIV vs. LAIV. Essentially, we tested all the AE case reports associated with TIV or LAIV independently against the whole VAERS database. Then the results of significantly enriched AEs in each group were identified using our combinatorial bioinformatics analysis pipeline. Our CODAE pipeline contains three methods for detection of true AE signals: PRR, Chi-square, and filtering based on the number of reports. After the significance of individual AEs associated with TIV or LAIV was identified, we compared the TIV and LAIV-enriched AEs through two ways. The first one is qualitative (i.e., presence or absence) comparison between the two AE lists. The second method is through quantitative comparison, *i.e.*, comparing the ratios of TIV (or LAIV)-associated AE case number over the total number of the same AE in the whole VAERS database. In the end, we used ontology-based methods to classify and compare significantly enriched AEs in each group.

We hypothesized that the AE differences in the two sets of recipients (TIV vs. LAIV) emerged from different immune-response pathways induced by each type of vaccine. Our study suggests that the combinatorial CODAE bioinformatics approach can overcome the complex challenges in public post-vaccination event record data. The strategy of this study resolves the issue of high-noise data, especially when these data contain high-value hidden knowledge that can be evaluated by robust statistical tests. It is crucial to identify background information, as some AEs are common to many vaccines. Because the number of reports in VAERS database is large (616,215 cases, 75 vaccines), background information is not sensitive to minor change or adjustment such as removing reports from one or two vaccines from the studied sample set. One novel feature of our combinatorial workflow (summarized in Figure 1) is its application in comparing two cohort sets of AEs. Another novelty of our approach is the use of the OAE for categorization of identified AEs. While this combinatorial workflow was applied specifically to VAERS data, the concept can also be adapted and applied to other questions in the Translational Informatics domain. Furthermore, the preliminary result in the form of flat list (*i.e.*, the simple text file of records) may be informative at an individual AE level. However, it is difficult to examine the flat list to identify the underlying biological systems when the system is composed of multiple interactions among multiple participating AEs. It is challenging to draw any connections between biological processes while the significant individual AE terms scatter across various different biological functions and systems. Examining these AEs based on their score rankings along with reorganizing results by their semantic similarity and functional relevance leads to a better representation of data that can overcome this issue. Analysis of alignment of semantic similarity to a reference structured controlled vocabulary is discussed below.

The results indicated that out of >37,000 TIV-associated AEs, 48 met the threshold for inclusion in the analysis, while of roughly 3,700 LAIV AEs, 68 met the threshold. Although this seems counter intuitive or surprising, TIV-associated AEs include two severe AEs (GBS and paralysis). Many other TIV-associated AEs are also related to neurological and muscular disorders, which can be considered as mild symptoms that can be further progressed to more severe symptoms including GBS and paralysis. GBS is classified as a syndrome that has indication of multiple symptoms. On the other hand, LAIV vaccination appears to induce many mild symptoms. No severe LAIV AEs that pass our thresholds has been detected.

The age patterns associated with the reports of GBS and other GBS-related disorders between patients immunized with TIV and LAIV are quite different (Figure 6). For TIV-associated GBS cases, the age range of 45–79 (34 years) has a high peak of > = 1 per 1,000 TIV AE case reports. The highest rate is approximately 2 cases per 1,000 TIV AE case reports at the age range of 55–69. For LAIV, the age ranges of 5–9 has slightly higher than 1 per 1,000 LAIV AE case reports, and the age range of 20–24 is associated with approximately 2 per 1,000 LAIV AE case reports. It appears that TIV is associated with GBS in a longer period of time and primarily occurs in adult and senior age, and LAIV-associated GBS primarily occurs in young age. Based on these observations, to better prevent GBS, it might be a good strategy to use TIV for young age patients and LAIV for adults and seniors.

Incidents of serious AEs are not always easy to detect in terms of population statistics as they may require a long period of observation. Therefore, the detection and confirmation of such incidents can be inconclusive or take a long time. Examples of time-consuming observations of vaccine post-marketing AEs include GBS after 1976 Swine Flu to 2009 A/H1N1 influenza vaccine campaigns [1], [19], anthrax vaccine adverse events (VAEs) studied from 1990 to 2007 [30], and 1990–2007 measles vaccine adverse effects studied in the Ivory Coast [31]. Although the 1976 incidence of GBS following Swine Flu vaccination was detected in real time, debate and discussion of the incidence remained inconclusive.

One interesting finding from this study was the occurrence of GBS in TIV recipients. There have been many controversial results with regard to the post influenza vaccination incidents, whether or not influenza vaccines induce GBS in the recipients. When considering specific subgroups of influenza vaccines (TIV versus LAIV), our analysis suggests that compared to LAIV, TIV is more strongly correlated with GBS (Table 3). Haber et al. concluded that the occurrence of GBS in influenza vaccine recipients was merely temporal association, and the causal association was not implicated with any solid evidence [1]. Furthermore, Haber et al. had challenged the study of Souayah et al. [32] that used the VAERS dataset by pointing out the VAERS limitation due to lack of standardized case follow-up. Haber et al. also argued that influenza vaccine-associated GBS incidence should be determined by influenza season rather than calendar year [33]. After a careful examination of the data, we found that pooling the entire VAERS dataset with our methodology could overcome the issues of omitted data or reporting intervals. Whether or not the reporting interval was based on the season or calendar year, overall incidence rate was not dependent on any one particular year or season. The number of post-influenza-vaccine GBS confirmed by neurologists in VAERS (1995–2003) as investigated by Haber et al. was observed to be 82%. This observation, when combined with additional data that became available in the later years, was still statistically significant as shown in a larger dataset such as the dataset used in this study. Souayah et al.'s study in 2009 remained firm in their conclusion of influenza vaccine-associated GBS with significant incidence rate [34]. Evans et al. also associated GBS and rare adverse events with influenza vaccine by conducting a comparative study of the novel influenza (swine flu) prepandemic data in 2009 to 1976 National influenza Immunization Program data [19]. Our study found that Souayah's and Evans' GBS association to influenza vaccines held true only when considering TIV, not LAIV. Furthermore, in a recent study by Moro et al., severe adverse events including GBS were implicated in the TIV high-dose recipients [35]. To the best of our knowledge, our systematic comparative study is the first to suggest that severe adverse events included GBS are more likely to be associated with trivalent (killed) inactivated influenza vaccine (TIV), but not live attenuated influenza vaccine (LAIV) or monovalent inactivated influenza vaccine.

As indicated in our meta-analysis, six out of 19 influenza vaccine-associated GBS reports show increased incidence rate of GBS ([36], [37], [38], [39], [40], [41]) (Table 4). In all these five studies that concluded the association between GBS and influenza vaccines, trivalent inactivated influenza vaccine (TIV) vaccination, instead of monovalent inactivated influenza vaccine (MIV) administration, was used. This phenomenon suggests no detected association of GBS to MIV vaccination. It is likely that the mix of different inactivated influenza strains in the TIV may increase the chance of obtaining GBS. However, these studies were usually based on individual case investigation with a relatively small cohort. Further investigation on the subject of monovalent versus trivalent inactivated influenza vaccines as the trigger of post-immunization GBS is required before conclusion can be made.

Even though the safety of TIVs is generally accepted at the population level, our analysis points towards LAIV as an alternative immunization that is less likely for the recipient to develop severe AEs such as GBS or paralysis. However, although the number of reported SAE cases associated with LAIV is very small that GBS and paralysis were not statistically enriched in LAIV group, the occurrences of LAIV-associated SAEs should still be investigated carefully. While GBS and paralysis (as categorized to be severe adverse events) were statistically enriched in the TIV group, for further study, the weighted-AE scoring method should be applied in future studies to properly address the issue of SAEs. All SAEs should automatically rank high in the significance of AE for both cohorts (TIV and LAIV).

Utilizing data from Vaccine Safety Datalink project, Lee et al. conducted a weekly sequential analysis of potential influenza vaccine adverse events from 9.2 million members in eight U.S. medical care organizations from November 2009 to April 2010 [22]. Both trivalent and monovalent seasonal killed and live attenuated influenza vaccines over a long observation period were examined. In total, 15 cases of GBS from 1,345,663 monovalent killed influenza vaccine (MIV)-vaccinated individuals were identified following MIV administration, 23 out of 2,741,150 cases after TIV, and zero out of 157,838 cases after LAIV. This study found that the GBS incidence after TIV administration was slightly lower than that after MIV administration. The incidence rates of GBS after both trivalent and monovalent killed influenza vaccines was approximately 1 in 100,000, which was not considered as statistically significant signals for GBS [22]. This study has also been included in our meta-analysis (Table 4). The result of this study does not change our meta-analysis conclusion that GBS was associated with TIV rather than MIV vaccination. Interestingly, zero cases of GBS were identified in LAIV-vaccinated patients, supporting our conclusion that LAIV may be safer than TIV in terms of induction of GBS vaccine adverse events. However, the sample size (157,838) of LAIV-vaccinated patients is relatively low compared to MIV- or TIV-vaccinated patients in their study. Our conclusion was drawn by combinatorial statistical analysis of VAERS case report data. Using all available VAERS case report data, our combinatorial method compares TIV- or LAIV-associated vaccine adverse events (VAEs) with VAEs associated with other vaccines. Our results suggest statistically significant association between TIV and GBS and paralysis, while LAIV shows no statistical evidence of correlation to GBS. Further studies to verify our conclusion of the higher safety of LAIV over TIV in terms of GBS induction are required.

One novel finding from our combinatorial bioinformatics analysis of influenza vaccine adverse events is that beside the relatively higher reporting rate of GBS and paralysis severe adverse event (SAE) cases associated with TIV than LAIV, TIV vaccination was also associated with a set of other mild neurological and muscular adverse events (*e.g.*, paraesthesia, hyporeflexia, musculoskeletal pain, and neuralgia) (Table 1). Although these symptoms are not considered SAEs, it is likely that these symptoms are signals of potential future GBS and paralysis SAEs due to their common neurological and muscular roots. The non-severe symptoms in some healthier patients may suggest signs of severe symptoms in other weaker patients. We hypothesize that the molecular interaction networks underlying these neurological and muscular adverse events, whether it is severe (*i.e.*, GBS and paralysis) or non-severe (*i.e.*, paraesthesia and hyporeflexia), are the same or similar at least at the early stage of these adverse events. By exploring these interaction networks, we can potentially identify the mechanisms of severe VAEs.

Further analysis of TIV- and LAIV-induced AEs by reorganizing into an ontological structure with reference to other community-accepted ontologies reveals certain challenges that need to be properly addressed. We have clustered AEs of each group of vaccines to COSTART (1995) (the foundation vocabulary that MedDRA was built upon) with an embedded hierarchical structure available on BioPortal (http://bioportal.bioontology.org/visualize/40390). We found that the COSTART hierarchical structure might not be a suitable term reorganization reference as the COSTART/MedDRA structure lacked a specific definition on which the aspect of this hierarchical tree was based. It was not clear if the hierarchy was defined by biological processes, or anatomy of the body. Hypothetically, COSTART/MedDRA is a comprehensive dictionary of adverse event descriptors; however, it was not created for the purpose of computation and the structure organization may not be fully equipped for ontological machine processing. Many concepts in COSTART listed synonyms that were not true synonyms. For example, in COSTART, sinus headache was defined to be synonymous to headache, and infection upper respiratory was defined to be synonymous to infection. Many examples of this kind of synonym error occur throughout the COSTART hierarchy. Another major issue in using COSTART as an ontological reference was that COSTART contained duplicate classes that caused ambiguity in many situations. For example, ear disorder was a child under a parent class of the same class name ear disorder, hemorrhage was a child of parent class haemorrhage [same word], and hypotension was a child of parent class shock syndrome which was, in turn, a sibling class of another concept that also has class identifier of hypotension. In an improvement of COSTART that results in MedDRA (version 12, released on 03/01/2009), terms are reorganized in a more comprehensive hierarchical structure, but further issues of one asserted class falling under multiple asserted parent nodes, or ambiguous synonym listings remain problematic. Examples of these classes can be found in class properties of Migraine, Migraine headache, Sinus headache, and other AEs throughout MedDRA (Figure S3).

We then explored another clinical ontology of SNOMED Clinical Terms (Version 07/31/2010) to find an alternative for AE term reorganization for the purpose of recognizing AEs based on biological relevance (Figure S4). We found that, while SNOMED CT was thoroughly defined with the most detailed information of anatomical and physiological description, this ontology still may not be the best alternative for such purposes. The comprehensive organization of terms in SNOMED CT resulted in a structure that did not provide an apparent clustering for term recognition based on biological process, because classes at the individual AE level (leaf nodes) were scattered across the ontology due to the nature of very detailed parental subclasses.

From the investigation of MedDRA and SNOMED CT as a reference controlled vocabulary, neither suited the purpose for such reference. Although MedDRA may not be the best nomenclature system for the purpose of AE reporting, it has been referenced in VAERS for many years. Therefore, to be able to mine for discovery within VAERS records, we must find methods to process and interpret VAERS data in an efficient way that discovers the knowledge embedded within it. Also, sometimes, MedDRA terms that are reported in VAERS fall into many semantic types in SNOMED CT, namely Body structure, Clinical finding, Procedure, Special concept, or Qualifier value. Such terms are within the same semantic type and there are also sub-structures that may further divide MedDRA terms into many separate groups. One example scenario that appeared in this situation is how Edema was described and categorized on the two ontologies. In SNOMED, Edema is a Clinical finding while Edema of pharynx is a child of Disorder characterized by edema, Disorder characterized by edema is a subclass of Disease, while Disease is a Clinical finding. This, in turn, resulted in Disease that was a sibling of class Edema while containing a child of a child of class Edema of pharynx. This separation by different semantic types occurred frequently in SNOMED-CT.

In this study, we have shown that the application of OAE can provide insights into underlying processes that may be overlooked or hard to detect without efficient prior-knowledge structural hierarchy (Figure 3 and Figure S2). Our combinatorial bioinformatics approach integrates different biostatistics analysis methods with ontology-based AE term classification. This method can also be modified to answer adverse event questions in different areas, for example, drug adverse events. It is noted that current study only used a part of the OAE features. Different from MedDRA and SNOMED-CT that represent adverse event outcomes, the OAE targets the representation of the whole process starting from the medical intervention (*e.g.*, vaccination) and ends with the discovery of the adverse event outcomes. Therefore, OAE provides a platform to examine in detail all the variables that can affect the results, such as patient age and sex, vaccination dose and route, and time interval between vaccination and the outcome of symptoms. Different from the original version of the Adverse Event Ontology (AEO) [15], the term “adverse event” defined in current OAE does not assume causal relation between an adverse event outcome and a medical intervention. The causal relation is now defined in an OAE term “causal adverse event”. One major task of the OAE research is to identify or predict the causal association based on various datasets including clinical adverse event reports.

Gleaning and cleaning *real-world* clinical data with this approach also introduces a novel hypothesis generator tool to aid translational informatics as the results are supported by statistical evaluation and validation of the findings. The method is designed to be discovery-driven rather than the traditional research hypothesis-driven approach. Two possible hypotheses that are derived from this post-vaccination adverse event investigation could be: (1) Hypothesis 1: TIV induces the occurrence of GBS that may be explained by the trigger in behavioral & neurological processes due to their possible shared gene interaction networks, and (2) Hypothesis 2: LAIV is more likely to trigger respiratory inflammatory response than TIV due to its mode of administration.

Conclusions from this study speak to the interest of personalized medicine of individuals. As a recent study by Liang et al. indicates the occurrence of GBS as below the background rate of severe adverse event induced by influenza vaccine [42], those observations were made on the whole population of influenza vaccine recipients including the majority who did not develop any major post-vaccination complication. Our study, in contrast, focuses on the sub-population of those whose cases have been submitted to VAERS as having a post-vaccination complication. This difference in the focused population group may lead to the hypothesis as to which molecular or genetic variation of the person can cause the occurrences of influenza vaccine-induced severe AEs.

## Supporting Information

Figure S1 **Signal curves to determine the data cutoff for TIV and LAIV analysis.** (A) Cutoff signal curve for TIV cutoff signal curve. (B) Cutoff signal curve for LAIV cutoff signal curve. Rendering and visualization of this plot was based on the adjusted display in MS Excel 2011 version 14.1.4. Some AE labels, though existed in dataset, were omitted on the plot.
(PDF)

Figure S2 **Classification of TIV- and LAIV-enriched vaccine adverse events using OAE.** TIV- and LAIV-enriched vaccine adverse event terms (MedDRA terms) identified in this study were mapped to OAE terms. The hierarchical structure of OAE was used to identify the parent terms and classify these of these adverse event (AE) terms.
(PDF)

Figure S3 **Classification of TIV- and LAIV-enriched vaccine adverse events using MedDRA.** TIV- and LAIV-enriched vaccine adverse event terms (MedDRA terms) identified in this study were classified using the hierarchical structure of MedDRA.
(PDF)

Figure S4 **Classification of TIV- and LAIV-enriched vaccine adverse events using SNOMED-CT.** TIV- and LAIV-enriched vaccine adverse event terms (MedDRA terms) identified in this study were mapped to SNOMED-CT terms. The hierarchical structure of SNOMED-CT was used to classify these terms.
(PDF)

Table S1 **Calculation of PRRs for vaccine adverse events.** Background information of Proportional Report Ratio calculation to determine signals of association of a vaccine to an AE.
(PDF)
