# Supplementary material for: Ontology-Based Combinatorial Comparative Analysis of Adverse Events Associated with Killed and Live Influenza Vaccines
Source: PLoS One. 2012 Nov 28;7(11):e49941. doi: 10.1371/journal.pone.0049941 (PMC3509157; doi:10.1371/journal.pone.0049941)
Supplement: Figure S1 — Signal curves to determine the data cutoff for TIV and LAIV analysis. (A) Cutoff signal curve for TIV cutoff signal curve. (B) Cutoff signal curve for LAIV cutoff signal curve. Rendering and visualization of this plot was based on the adjusted display in MS Excel 2011 version 14.1.4. Some AE labels, though existed in dataset, were omitted on the plot. (PDF) [file pone.0049941.s001.pdf]

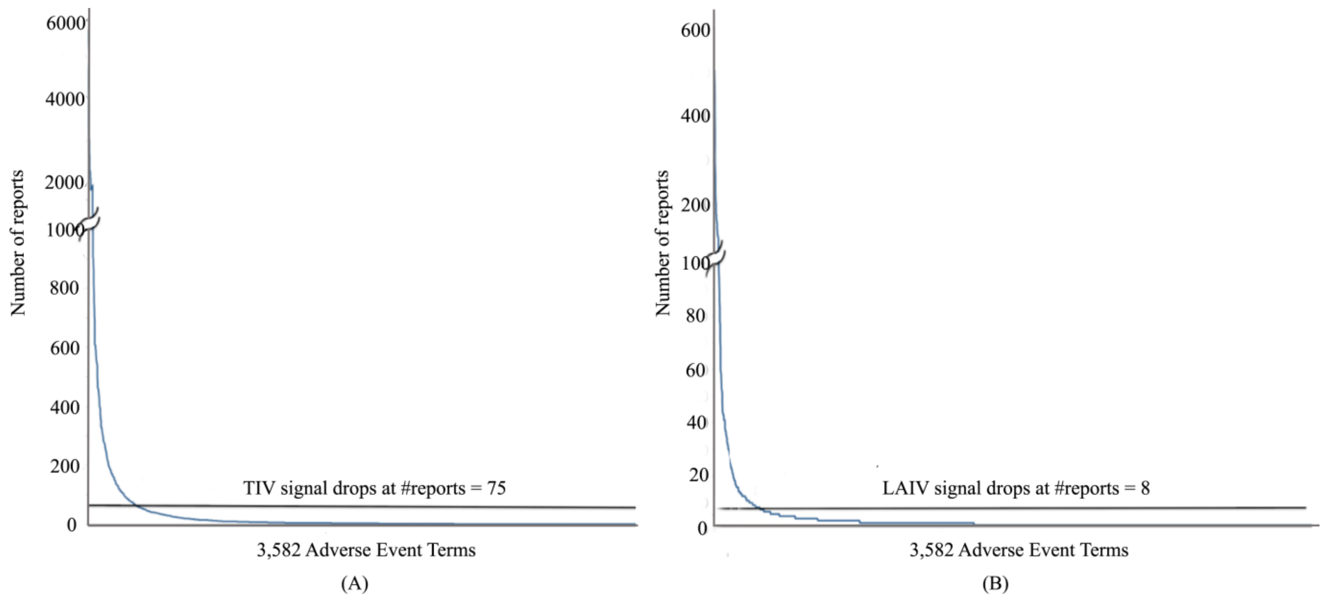

Supporting Figure S1. Signal curves to determine the data cut-off for TIV and LAIV analysis. (A) Cut-off signal curve for TIV cut-off signal curve. (B) Cut-off signal curve for LAIV cut-off signal curve. Rendering and visualization of this plot was based on the adjusted display in MS Excel 2011 version 14.1.4. Some AE labels, though existed in dataset, were omitted on the plot.
