# Supplementary material for: Ontology-Based Combinatorial Comparative Analysis of Adverse Events Associated with Killed and Live Influenza Vaccines
Source: PLoS One. 2012 Nov 28;7(11):e49941. doi: 10.1371/journal.pone.0049941 (PMC3509157; doi:10.1371/journal.pone.0049941)
Supplement: Figure S2 — Classification of TIV- and LAIV-enriched vaccine adverse events using OAE. TIV- and LAIV-enriched vaccine adverse event terms (MedDRA terms) identified in this study were mapped to OAE terms. The hierarchical structure of OAE was used to identify the parent terms and classify these of these adverse event (AE) terms. (PDF) [file pone.0049941.s002.pdf]

TIV

- Thing
  - 'bodily process'
    - 'pathological bodily process'
      - 'KIV-induced adverse event'
        - 'AE with an outcome of lab test abnormal'
          - 'electromyogram abnormal AE'
        - 'behavior and neurological AE'
          - 'dysarthria AE'
          - 'movement disorder AE'
            - 'paralysis AE'
            - 'reflexes decreased AE'
              - 'hyporeflexia AE'
            - 'sensation of heaviness AE'
            - 'throat tightness AE'
          - 'sensory capability AE'
            - 'chills AE'
            - 'hypoaesthesia AE'
              - 'hypoaesthesia facial AE'
              - 'hypoaesthesia oral AE'
            - 'pain AE'
              - 'chest pain AE'
              - 'musculoskeletal pain AE'
              - 'neck pain AE'
              - 'neuralgia AE'
              - 'pain in extremity AE'
              - 'shoulder pain AE'
            - 'palpitation AE'
            - 'sensation of heaviness AE'
            - 'skin burning sensation AE'
        - 'cardiovascular disorder AE'
          - 'abnormal blood pressure AE'
            - 'hypertension AE'
          - 'abnormal heartbeat AE'
            - 'increased heart rate AE'
          - 'haematoma AE'
            - 'injection-site haematoma AE'
        - 'digestive system AE'
          - 'dry mouth AE'
          - 'dysphagia AE'
        - 'eye disorder AE'
          - 'eye discharge AE'
          - 'eye irritation AE'
        - 'homeostasis AE'
          - 'abnormal fluid regulation AE'
            - 'edema AE'
              - 'local swelling AE'
              - 'pharyngeal edema AE'
              - 'tongue edema AE'
        - 'musculoskeletal system AE'
          - 'muscle adverse event'
            - 'muscle spasm AE'
              - 'laryngospasm AE'
            - 'muscular weakness AE'
        - 'nervous system AE'
          - 'abnormal cerebrospinal fluid production AE
            - 'CSF protein increased AE'
          - 'Guillain-Barre syndrome AE'
          - 'mobility decreased AE'
            - 'injected limb mobility decreased AE'
            - 'joint range of motion decreased AE'
        - 'respiratory system AE'
          - 'abnormal respiration AE'
            - 'dypsnoea AE'
        - 'skin adverse event'
          - 'hot flush AE'
          - 'pruritus AE'
            - 'eye pruritus AE'
          - 'skin discoloration AE'
            - 'flushing AE'
  - 'medical intervention'
    - 'incorrect dose administration'
      - 'accidental overdose in medical intervention'

LAIV

- Thing
  - 'bodily process'
    - 'pathological bodily process'
      - 'LAIV-induced adverse event'
        - 'AE with an outcome of lab test abnormal'
          - 'X-ray abnormal AE'
            - 'chest X-ray abnormal AE'
          - 'blood cell lab test abnormal AE'
            - 'blood creatine phosphokinase increased AE'
            - 'influenza serology positive AE'
            - 'neutrophil percentage increased AE'
          - 'computerised tomogram abnormal AE'
          - 'electrocardiogram abnormal AE'
          - 'nuclear magnetic resonance imaging brain abnormal AE'
          - 'urine analysis abnormal AE'
            - 'urine ketone body present AE'
        - 'behavior and neurological AE'
          - 'fatigue AE'
          - 'movement disorder AE'
            - 'paralysis AE'
              - 'VIIth nerve paralysis AE'
          - 'sensory capability AE'
            - 'abdominal discomfort AE'
            - 'burning sensation AE'
            - 'pain AE'
              - 'abdominal pain AE'
                - 'abdominal pain upper AE'
              - 'ear pain AE'
              - 'headache AE'
                - 'migraine AE'
                - 'sinus headache AE'
        - 'cardivasular disorder AE'
          - 'hemorrhage AE'
            - 'epistaxis AE'
        - 'digestive system AE'
          - 'dry throat AE'
          - 'retching AE'
        - 'eye disorder AE'
          - 'eye irritation AE'
          - 'photophobia AE'
        - 'gustatory system AE'
          - 'throat irritation AE'
        - 'homeostasis AE'
          - 'abnormal fluid regulation AE'
            - 'edema AE'
              - 'eyelid edema AE'
              - 'face edema AE'
        - 'infection AE'
          - 'croup infection AE'
        - 'injury and procedural complication AE'
          - 'pregnancy test positive AE'
        - 'respiratory system AE'
          - 'asthma AE'
          - 'nasal congestion AE'
            - 'sinus congestion AE'
          - 'nasal discomfort AE'
          - 'pneumonia AE'
            - 'lobar pneumonia AE'
          - 'postnasal drip AE'
          - 'respiratory system inflammation AE'
            - 'bronchitis AE'
            - 'nasopharyngitis AE'
            - 'sinus inflammation AE'
              - 'sinusitis AE'
          - 'respiratory tract congestion AE'
          - 'rhinorrhoea AE'
          - 'sneezing AE'
          - 'upper respiratory tract infection AE'
          - 'wheezing AE'
            - 'stridor AE'
        - 'skin adverse event'
          - 'pruritus AE '
            - 'pruritus generalised AE'
          - 'rash AE'
            - 'pustula rash AE'
          - 'skin discoloration AE'
            - 'purpura AE'
              - 'Henoch-Schonlein purpura AE'
        - 'social behavior AE'
          - 'acitivities of daily living impaired AE'
  - 'medical intervention'
    - 'accidental exposure in medical intervention'
    - 'drug administration'
      - 'drug exposure during pregnancy'
      - 'errored drug administration'
        - 'expired drug administration'
        - 'inappropriate schedule of drug administration'
    - 'incorrect dose administraiton '
      - 'underdose administration in medical intervention'
    - vaccination
      - 'errored vaccination'

Supporting Figure S2. Classification of TIV- and LAIV-specific VAEs using OAE.

It is noted that all AEs in OAE are pathological bodily processes. An AE process in OAE starts with a medical intervention (*e.g.*, vaccination) and ends with the discovery of an AE outcome including a symptom (*e.g.*, pain), sign (*e.g.*, increased level of glucose), or process (*e.g.*, bacterial infection). This is different from MedDRA and SNOMED-CT that focus on only the adverse event outcomes. This is the reason why the names of our OAE AE terms usually include the letters "AE" in the end, for example, "pain AE", representing "pain adverse event". In this example, the symptom "pain" is the outcome, and "pain adverse event" is a pathological bodily process that starts with a medical intervention (*e.g.*, vaccination) and ends with the outcome of pain.
