# Supplementary material for: Ontology-Based Combinatorial Comparative Analysis of Adverse Events Associated with Killed and Live Influenza Vaccines
Source: PLoS One. 2012 Nov 28;7(11):e49941. doi: 10.1371/journal.pone.0049941 (PMC3509157; doi:10.1371/journal.pone.0049941)
Supplement: Figure S3 — Classification of TIV- and LAIV-enriched vaccine adverse events using MedDRA. TIV- and LAIV-enriched vaccine adverse event terms (MedDRA terms) identified in this study were classified using the hierarchical structure of MedDRA. (PDF) [file pone.0049941.s003.pdf]

TIV

- Thing (MedDRA Version 12.0 03/01/2009)
  - 'Cardiac disorders'
    - 'Cardiac disorder signs and symptoms'
      - 'Cardiac signs and symptoms NEC'
        - Palpitations
  - 'Eye disorders'
    - 'Occular infections, irritations and inflammations'
      - 'Ocular infections, inflammations and associated manifestations'
        - 'Eye discharge'
        - 'Eye irritation'
        - 'Eye pruritus'
  - 'Gastrointestinal disorders'
    - 'Gastrointestinal signs and symptoms'
      - 'Gastrointestinal signs and symptoms NEC'
        - Dysphagia
    - 'Salivary gland conditions'
      - 'Oral dryness and saliva altered'
        - 'Dry mouth'
    - 'Tongue conditions'
      - 'Tongue signs and symptoms'
        - 'Swollen tongue'
        - 'Tongue oedema'
  - 'General disorders and administration site conditions'
    - 'Administration site reactions'
      - 'Injection site reactions'
        - 'Injected limb mobility decreased'
        - 'Injection site haematoma'
    - 'General system disorders NEC'
      - 'Feelings and sensations NEC'
        - Chills
        - 'Feeling cold'
      - 'General signs and symptoms NEC'
        - Flushing
        - 'Hot flush'
        - 'Local swelling'
        - 'Mobility decreased'
      - 'Pain and discomfort NEC'
        - 'Chest pain'
        - Pain
  - 'Immune system disorders'
    - 'Autoimmune disorders'
      - 'Autoimmune disorder NEC'
        - 'Guillain-Barre syndrome'
  - 'Injury, poisoning and procedural complications'
    - 'Medication errors'
      - Overdose
        - 'Accidental overdose'
  - Investigations
    - 'Cardiac and vascular investigations (excl enzyme tests)'
      - 'Vascular tests NEC (incl blood pressure)'
        - 'Blood pressure increased'
    - 'Heart rate and pulse investigations'
      - 'Heart rate increased'
    - 'Neurological, special senses and psychiatric investigations'
      - 'Neurologic diagnostic procedures'
        - 'Electromyogram abnormal'
  - 'Musculoskeletal and connective tissue disorders'
    - 'Joint disorders'
      - 'Joint related signs and symptoms'
        - 'Joint range of motion decreased'
    - 'Muscle disorders'
      - 'Muscle weakness conditions'
        - 'Muscular weakness'
    - 'Musculoskeletal and connective tissue disorders NEC'
      - 'Musculoskeletal and connective tissue pain and discomfort'
        - 'Musculoskeletal pain'
        - 'Neck pain'
        - 'Pain in extremity'
      - 'Musculoskeletal and connective tissue signs and symptoms NEC'
        - 'Sensation of heaviness'
  - 'Nervous system disorders'
    - 'Movement disorders (incl parkinsonism)'
      - 'Paralysis and paresis (excl cranial nerve)'
        - Paralysis
    - 'Neurological disorders NEC'
      - 'Abnormal reflexes'
        - Hyporeflexia
      - 'Paraesthesias and Dysaesthesias'
        - Paraesthesia
      - 'Sensory abnormalities NEC'
        - Hypoaesthesia
        - Neuralgia
  - 'Psychiatric disorders'
    - 'Communication disorders and disturbances'
      - 'Speech articulation and rhythm disturbances'
        - Dysarthria
    - 'Psychiatric and behavioural symptoms NEC'
      - 'Psychiatric symptoms NEC'
        - 'Throat tightness'
  - 'Respiratory, thoracic and mediastinal disorders'
    - 'Respiratory disorders NEC'
      - 'Breathing abnormalities'
        - Dyspnoea
    - 'Upper respiratory tract disorders (excl infections)'
      - 'Laryngeal spasm, oedema and obstruction'
        - Laryngospasm
      - 'Pharyngeal disorders (excl infections and neoplasms)'
        - 'Pharyngeal oedema'
  - 'Skin and subcutaneous tissue disorders'
    - 'Epidermal and dermal conditions'
      - 'Dermal and epidermal conditions NEC'
        - 'Skin burning sensation'
  - 'Vascular disorders'
    - 'Vascular hypertensive disorders'
      - 'Vascular hypertensive disorders NEC'
        - Hypertension

LAIV

- Thing (MedDRA Version 12.0 03/01/2009)
  - 'Immune system disorders'
    - 'Immune disorders NEC'
      - Vasculitides
        - 'Henoch-Schonlein purpura '
  - 'Blood and lymphatic system disorders'
    - 'Coagulopathies and bleeding diatheses (excl thrombocytopenic)'
      - 'Purpuras (excl thrombocytopenic)'
        - 'Henoch-Schonlein purpura '
  - 'Social circumstances'
    - 'Lifestyle issues'
      - 'Disability issues'
        - 'Impaired work ability'
        - 'Activities of daily living impaired'
  - 'Pregnancy, puerperium and perinatal conditions'
    - 'Pregnancy, labour, delivery and postpartum conditions'
      - 'High risk pregnancies'
        - 'Drug exposure during pregnancy'
  - 'infections and infestations'
    - 'Viral infectious disorders'
      - 'Parainfluenza viral infections'
        - 'Croup infectious'
  - 'Skin and subcutaneous tissue disorders'
    - 'Skin vascular abnormalitites'
      - 'Purpura and related conditions'
        - 'Henoch-Schonlein purpura '
    - 'Angioedema and urticaria'
      - Angioedemas
        - 'Eyelid oedema'
    - 'Epidermal and dermal conditions'
      - 'Pustular conditions'
        - 'Rash pustular'
      - 'Pruritus NEC'
        - 'Pruritus generalised'
      - 'Dermal and epidermal conditions NEC'
        - 'Swelling face'
  - 'Eye disorders'
    - 'Vision disorders'
      - 'Visual disorders NEC'
        - 'Visual impairment'
    - 'Ocular infections, irritations and inflammations'
      - 'Ocular infections, inflammations and associated manifestations'
        - 'Eye irritation'
    - 'Ocular sensory symptoms NEC'
      - 'Ocular sensation disorders'
        - Photophobia
  - 'Injury, poisoning and procedural complications'
    - 'Medication errors'
      - 'Medication errors NEC'
        - 'Vaccination error'
      - 'Medication errors due to accidental exposures'
        - 'Accidental exposure'
      - Maladministrations
        - 'Drug administration error'
        - Underdose
        - 'Inappropriate schedule of drug administration'
        - 'Expired drug administered'
  - 'Cardiac disorders'
    - 'Pericardial disorders'
      - 'Noninfectious pericarditis'
        - Pericarditis
  - 'Respiratory, thoracic and mediastinal disorders'
    - 'Bronchial disorders (excl neoplasms)'
      - 'Bronchospasm and obstruction'
        - asthma
        - Bronchospasm
    - 'Respiratory tract infections'
      - 'Upper respiratory tract infections NEC'
        - Nasopharyngitis
        - 'Upper respiratory tract infection'
        - Sinusitis
      - 'Lower respiratory tract infections NEC'
        - 'Lobar pneumonia'
        - Bronchitis
        - Pneumonia
      - 'Viral upper respiratory tract infections'
        - 'Croup infectious'
    - 'Upper respiratory tract disorders (excl infections)'
      - 'Larygeal spasm, oedema and obstruction'
        - Stridor
      - 'Paranasal sinus disorders (excl infections and neoplasms)'
        - 'Sinus congestion'
      - 'Nasal congestion and inflammations'
        - 'Nasal congestion'
      - 'Nasal disorders NEC'
        - Epistaxis
    - 'Respiratory disorders NEC'
      - 'Respiratory tract disorders NEC'
        - 'Respiratory tract congestion'
      - 'Upper respiratory tract signs and symptoms'
        - 'Postnasal drip'
        - Sneezing
        - Rhinorrhoea
        - 'Throat irritation'
        - 'Dry throat'
        - 'Sinus headache'
  - 'Vascular disorders'
    - 'Vascular haemorrhagic disorders'
      - 'Bruising, ecchymosis and purpura'
        - 'Henoch-Schonlein purpura'
      - 'Haemorrhages NEC'
        - 'Epistaxis '
    - 'Vascular disorders NEC'
      - 'Cerebrovascular and spinal vascular disorders NEC'
        - Migraine
  - 'Ear and labyrinth disorders'
    - 'Aural disorders NEC'
      - 'Ear disorder NEC'
        - 'Ear pain'
  - 'Gastrointestinal disorders'
    - 'Oral soft tissue condtions'
      - 'Throat irritations '
    - 'Gastrointestinal signs and symptoms'
      - 'Nausea and vomiting symptoms'
        - Retching
      - 'Gastrointestinal signs and symptoms NEC'
        - 'Abdominal discomfort'
      - 'Gastrointestinal and abdominal pains (excl oral and throat)'
        - 'Abdominal pain upper'
  - 'General disorders and administration site conditions'
    - 'Therapeutic and nontherapeutic effects'
      - 'Therapeutic and nontherapeutic responses'
        - 'Immunisation reaction'
    - 'General system disorders NEC'
      - 'Gait disturbances'
        - Ataxia
      - 'Asthenic conditions'
        - Fatigue
  - 'Nervous system disorders'
    - 'Cranial nerve disorders (excl neoplasms)'
      - 'Facial cranial nerve disorders'
        - 'Facial paresis'
        - 'VIIth nerve paralysis'
    - 'Neurological disorders NEC'
      - 'Cerebellar coordination and balance disturbances'
        - 'Ataxia '
      - 'Paraesthesias and dysaesthesias'
        - 'Burning sensation'
    - Headaches
      - 'Migraine headaches'
        - 'Migraine '
      - 'Headaches NEC'
  - Investigations
    - 'Endocrine investigations (incl sex hormones)'
      - 'Reproductive hormone analyses'
        - 'Pregnancy test positive'
    - 'Cardiac and vascular investigations (excl enzyme tests)'
      - 'ECG investigations'
        - 'Electrocardiogram abnormal'
    - 'Enzyme investigations NEC'
      - 'Skeletal and cardiac muscle analyses'
        - 'Blood creatine phosphokinase increased'
    - 'Haematology investigations (incl blood groups)'
      - 'White blood cell analyses'
        - 'Lymphocyte percentage decreased'
        - 'Neutrophil percentage increased'
    - 'Investigations, imaging and histopathology procedures NEC'
      - 'Imaging procedures NEC'
        - 'Computerised tomogram abnormal'
    - 'Metabolic, nutritional and blood gas investigations'
      - 'Carbohydrate tolerance analyses (incl diabetes)'
        - 'Blood glucose increased'
      - 'Metabolism tests NEC'
        - 'Urine ketone body present'
    - 'Microbiology and serology investigations'
      - 'Virus identification and serology'
        - 'Influenza serology positive'
    - 'Neurological, special senses and psychiatric investigations'
      - 'Central nervous system imaging procedures'
        - 'Nuclear magnetic resonance imaging brain abnormal'
    - 'Renal and urinary tract investigations and urinalyses'
      - 'Urinalysis NEC'
        - 'Urine analysis abnormal'
    - 'Respiratory and pulmonary investigations (excl blood gases)'
      - 'Respiratory tract and thoracic imaging procedures'
        - 'Chest X-ray abnormal'

Supporting Figure S3. Classification of TIV- and LAIV-specific vaccine adverse events using MedDRA.
