# Supplementary material for: Ontology-Based Combinatorial Comparative Analysis of Adverse Events Associated with Killed and Live Influenza Vaccines
Source: PLoS One. 2012 Nov 28;7(11):e49941. doi: 10.1371/journal.pone.0049941 (PMC3509157; doi:10.1371/journal.pone.0049941)
Supplement: Figure S4 — Classification of TIV- and LAIV-enriched vaccine adverse events using SNOMED-CT. TIV- and LAIV-enriched vaccine adverse event terms (MedDRA terms) identified in this study were mapped to SNOMED-CT terms. The hierarchical structure of SNOMED-CT was used to classify these terms. (PDF) [file pone.0049941.s004.pdf]

TIV

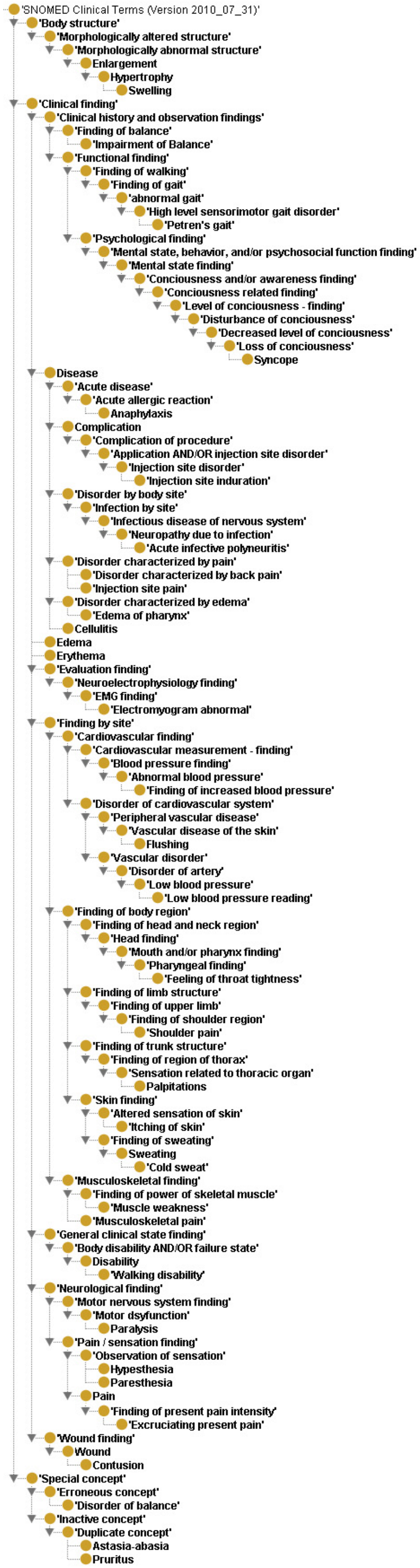

'SNOMED Clinical Terms (Version 2010_07_31)'
'Body structure'
'Morphologically altered structure'
'Morphologically abnormal structure'
Enlargement
Hypertrophy
Swelling
'Clinical finding'
'Clinical history and observation findings'
'Finding of balance'
'Impairment of Balance'
'Functional finding'
'Finding of walking'
'Finding of gait'
'abnormal gait'
'High level sensorimotor gait disorder'
'Petren's gait'
'Psychological finding'
'Mental state, behavior, and/or psychosocial function finding'
'Mental state finding'
'Conciousness and/or awareness finding'
'Conciousness related finding'
'Level of conciousness - finding'
'Disturbance of conciousness'
'Decreased level of conciousness'
'Loss of conciousness'
Syncope
Disease
'Acute disease'
'Acute allergic reaction'
Anaphylaxis
Complication
'Complication of procedure'
'Application AND/OR injection site disorder'
'Injection site disorder'
'Injection site induration'
'Disorder by body site'
'Infection by site'
'Infectious disease of nervous system'
'Neuropathy due to infection'
'Acute infective polyneuritis'
'Disorder characterized by pain'
'Disorder characterized by back pain'
'Injection site pain'
'Disorder characterized by edema'
'Edema of pharynx'
Cellulitis
Edema
Erythema
'Evaluation finding'
'Neuroelectrophysiology finding'
'EMG finding'
'Electromyogram abnormal'
'Finding by site'
'Cardiovascular finding'
'Cardiovascular measurement - finding'
'Blood pressure finding'
'Abnormal blood pressure'
'Finding of increased blood pressure'
'Disorder of cardiovascular system'
'Peripheral vascular disease'
'Vascular disease of the skin'
Flushing
'Vascular disorder'
'Disorder of artery'
'Low blood pressure'
'Low blood pressure reading'
'Finding of body region'
'Finding of head and neck region'
'Head finding'
'Mouth and/or pharynx finding'
'Pharyngeal finding'
'Feeling of throat tightness'
'Finding of limb structure'
'Finding of upper limb'
'Finding of shoulder region'
'Shoulder pain'
'Finding of trunk structure'
'Finding of region of thorax'
'Sensation related to thoracic organ'
Palpitations
'Skin finding'
'Altered sensation of skin'
'Itching of skin'
'Finding of sweating'
Sweating
'Cold sweat'
'Musculoskeletal finding'
'Finding of power of skeletal muscle'
'Muscle weakness'
'Musculoskeletal pain'
'General clinical state finding'
'Body disability AND/OR failure state'
Disability
'Walking disability'
'Neurological finding'
'Motor nervous system finding'
'Motor dysfunction'
Paralysis
'Pain / sensation finding'
'Observation of sensation'
Hypesthesia
Paresthesia
Pain
'Finding of present pain intensity'
'Excruciating present pain'
'Wound finding'
Wound
Contusion
'Special concept'
'Erroneous concept'
'Disorder of balance'
'Inactive concept'
'Duplicate concept'
Astasia-abasia
Pruritus

LAIV

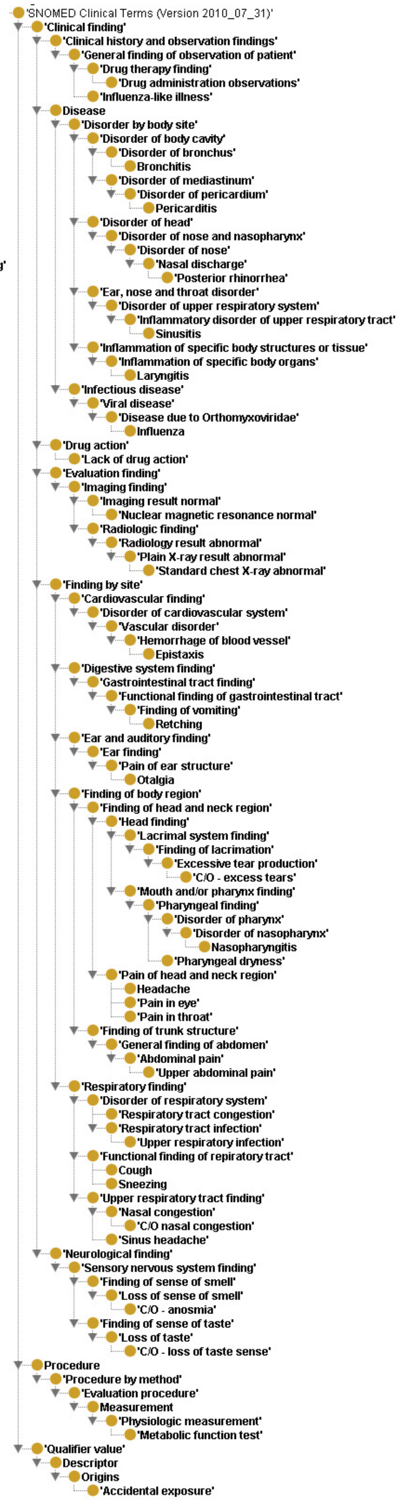

'SNOMED Clinical Terms (Version 2010_07_31)'
'Clinical finding'
'Clinical history and observation findings'
'General finding of observation of patient'
'Drug therapy finding'
'Drug administration observations'
'Influenza-like illness'
Disease
'Disorder by body site'
'Disorder of body cavity'
'Disorder of bronchus'
Bronchitis
'Disorder of mediastinum'
'Disorder of pericardium'
Pericarditis
'Disorder of head'
'Disorder of nose and nasopharynx'
'Disorder of nose'
'Nasal discharge'
'Posterior rhinorrhea'
'Ear, nose and throat disorder'
'Disorder of upper respiratory system'
'Inflammatory disorder of upper respiratory tract'
Sinusitis
'Inflammation of specific body structures or tissue'
'Inflammation of specific body organs'
Laryngitis
'Infectious disease'
'Viral disease'
'Disease due to Orthomyxoviridae'
Influenza
'Drug action'
'Lack of drug action'
'Evaluation finding'
'Imaging finding'
'Imaging result normal'
'Nuclear magnetic resonance normal'
'Radiologic finding'
'Radiology result abnormal'
'Plain X-ray result abnormal'
'Standard chest X-ray abnormal'
'Finding by site'
'Cardiovascular finding'
'Disorder of cardiovascular system'
'Vascular disorder'
'Hemorrhage of blood vessel'
Epistaxis
'Digestive system finding'
'Gastrointestinal tract finding'
'Functional finding of gastrointestinal tract'
'Finding of vomiting'
Retching
'Ear and auditory finding'
'Ear finding'
'Pain of ear structure'
Otalgia
'Finding of body region'
'Finding of head and neck region'
'Head finding'
'Lacrimal system finding'
'Finding of lacrimation'
'Excessive tear production'
'C/O - excess tears'
'Mouth and/or pharynx finding'
'Pharyngeal finding'
'Disorder of pharynx'
'Disorder of nasopharynx'
Nasopharyngitis
'Pharyngeal dryness'
'Pain of head and neck region'
Headache
'Pain in eye'
'Pain in throat'
'Finding of trunk structure'
'General finding of abdomen'
'Abdominal pain'
'Upper abdominal pain'
'Respiratory finding'
'Disorder of respiratory system'
'Respiratory tract congestion'
'Respiratory tract infection'
'Upper respiratory infection'
'Functional finding of repiratory tract'
Cough
Sneezing
'Upper respiratory tract finding'
'Nasal congestion'
'C/O nasal congestion'
'Sinus headache'
'Neurological finding'
'Sensory nervous system finding'
'Finding of sense of smell'
'Loss of sense of smell'
'C/O - anosmia'
'Finding of sense of taste'
'Loss of taste'
'C/O - loss of taste sense'
Procedure
'Procedure by method'
'Evaluation procedure'
Measurement
'Physiologic measurement'
'Metabolic function test'
'Qualifier value'
Descriptor
Origins
'Accidental exposure'

Supporting Figure S4. Classification of TIV- and LAIV-specific vaccine adverse events using SNOMED-CT.
