# Supplementary material for: Ontology-Based Combinatorial Comparative Analysis of Adverse Events Associated with Killed and Live Influenza Vaccines
Source: PLoS One. 2012 Nov 28;7(11):e49941. doi: 10.1371/journal.pone.0049941 (PMC3509157; doi:10.1371/journal.pone.0049941)
Supplement: Table S1 — Calculation of PRRs for vaccine adverse events. Background information of Proportional Report Ratio calculation to determine signals of association of a vaccine to an AE. (PDF) [file pone.0049941.s005.pdf]

Supporting Table S1. Calculation of PRRs for vaccine adverse events

	Vaccine(s) of interest	All other vaccines in VAERS
**Adverse event(s) of interest**	**a**	**b**
**All other adverse events**	**c**	**d**

Note: The proportional reporting ratio (PRR) statistical method calculates the proportions of specific adverse events for a vaccine (or a group of vaccines) of interest where the comparator is all other vaccines in the VAERS database.

Specifically, PRR = a/(a+c) divided by b/(b+d) in a two by two table (see above)

As shown in the formula, the denominator (comparator) is all other vaccines in the VAERS database. If some AEs occur much more frequently than others for the other vaccines in the general VAERS database, this will increase the number of the denominator, leading to deceased PRR value. This can be considered as background noise.

PRR is a valuable method to detect signals from spontaneous reporting data. A high PRR value (e.g., 3-5) suggests a need for detailed evaluation of the targeted adverse event. In contrast, a PRR value close to or less than one may prevent unnecessary effort in evaluating case series. Such a low PRR value is usually the consequence of background "noise" and does not truly represent a signal.

Reference:

Evans SJ, Waller PC, Davis S (2001) Use of proportional reporting ratios (PRRs) for signal generation from spontaneous adverse drug reaction reports. Pharmacoepidemiology and drug safety 10: 483-486.
